# The efficacy and safety of using prophylactic abdominal drainage after laparoscopic cholecystectomy: A randomized control trial

**DOI:** 10.1002/hsr2.2284

**Published:** 2024-08-08

**Authors:** Ahmad Y. Arnaout, Lama Kadoura, Ruqaya Masri, Yaman Nerabani, Abd Alazeez Atli, Khaled Arnaout, Ibrahim Arnaout, Adel Bsata, Hasan Kayali, Nehad Mahli, Ahmad Al‐Haj, Kusay Ayoub, Ammar Niazi, Ahmad Ghazal, Aleppo University Hospital Team, Ezeddin Dabbagh, Ola Hamdan, Abdallah Dabbit, Mohamad Zaher Shahrour, Wael Alkhaleel, Hasan Mohammad Alhasan, Nour Lbabidi, Hilal Mohammad Matar, Amer Zidany, Shergal Tammo, Mousa Ahmad Sifat

**Affiliations:** ^1^ Faculty of Medicine, Aleppo University Hospital University of Aleppo Aleppo Syrian Arab Republic; ^2^ Department of Surgery, Aleppo University Hospital University of Aleppo Aleppo Syrian Arab Republic; ^3^ Faculty of Medicine, Aleppo University Hospital University of Aleppo Aleppo Syrian Arab Republic; ^4^ Department of Surgery, Aleppo University Hospital University of Aleppo Aleppo Syrian Arab Republic

**Keywords:** laparoscopic cholecystectomy, prophylactic drainage, randomized control trial

## Abstract

**Background:**

The use of prophylactic drainage after laparoscopic cholecystectomy has been a routine practice for many years. However, the debate surrounding using it stems from conflicting evidence regarding its potential benefits and risks.

**Methods:**

Patients who underwent laparoscopic cholecystectomy from February 1, 2022, to November 30, 2022, at Aleppo University Hospital were enrolled according to our previously registered protocol (NCT05267860).

**Results:**

This study included 232 patients (117 in the drainage group [DG], and 115 in the non‐drainage group [NDG]). There was no statistical difference in the patients' main characteristics, comorbidities, and laboratory findings. The duration of the surgical operation in NDG (mean = 44.92, SD = 1.85) was shorter than in DG (mean 55.14, SD = 2.14), with (*p* = 0.039) statistically significant, which indicates that the use of the drainage led to a prolongation of the surgical operation. The total number of complicated cases reached 22 (9.48%) cases (DG = 9 vs. NDG = 13, *p* = 0.348) as follows: bleeding (*n* = 1) (DG = 1 vs. NDG = 0; *p* = 0.320), bile leak with no established bile duct injury (*n* = 1) (DG = 1 vs. NDG = 0; *p* = 0.320), wound infection (*n* = 12) (DG = 4 vs. NDG = 8; *p* = 0.443), urinary tract infection (*n* = 3) (DG = 0 vs. NDG = 3; *p* = 0.079), prolonged shoulder pain (*n* = 2) (DG = 0 vs. NDG = 2; *p* = 0.152), and acute pancreatitis (*n* = 1) (DG = 1 vs. NDG = 0; *p* = 0.144).

**Conclusion:**

Based on the results of our study, the use of prophylactic drainage was safe, but ineffective, as it did not improve the outcomes statistically significantly or worsen them, which is consistent with previous studies highlighting the need for personalized patient care in this setting.

## 1 INTRODUCTION

Laparoscopic cholecystectomy is a commonly performed surgical procedure for the treatment of gallbladder diseases, with millions of cases conducted worldwide each year. For many years, the use of prophylactic abdominal drainage after laparoscopic cholecystectomy has been a common practice. Historically, many medical professionals believed in the value of using drains post‐abdominal surgeries to eliminate intra‐abdominal collections like ascites, blood, bile, chyle, and pancreatic or intestinal juices, as advocated by renowned German surgeon Theodor Billroth.^1^, ^2^

However, recent advancements in surgical techniques and perioperative care have led to a reevaluation of the necessity and efficacy of routine drainage in this setting. The debate surrounding the use of prophylactic abdominal drainage after laparoscopic cholecystectomy stems from conflicting evidence regarding its potential benefits and risks. While some studies have suggested a possible reduction in postoperative complications with drain placement,^3^ others have indicated no significant difference in outcomes or even a potential increase in complications associated with drain use.^4^, ^5^ In fact, placing a routine drain may even be detrimental to patients undergoing laparoscopic cholecystectomy for noncomplicated benign gallbladder conditions,^6^ and noncomplicated open cholecystectomy procedures.^7^

Given the lack of consensus in the current literature, there is a critical need for well‐designed randomized controlled trials to provide more definitive evidence on the efficacy and safety of prophylactic abdominal drainage after laparoscopic cholecystectomy. This study aims to address this gap in knowledge by conducting a randomized controlled trial to evaluate the impact of prophylactic drain usage on postoperative outcomes, complications, and patient safety in the context of laparoscopic cholecystectomy.

## 2 METHODS

### 2.1 Participants and allocation

Patients who underwent laparoscopic cholecystectomy from February 1, 2022, to November 30, 2022, at Aleppo University Hospital were enrolled in the study after obtaining verbal consent and explaining the details of the procedure to them according to our previously registered protocol (NCT05267860) and following the Consolidated Standards of Reporting Trials (CONSORT) Guidelines.^8^

The inclusion criteria encompassed patients aged 18−90 who underwent laparoscopic cholecystectomy for any reason, did not necessitate a curative drain, and agreed to participate. Excluded were patients who underwent open cholecystectomy or required a curative drain due to surgery‐related complications.

Random allocation of the 232 participants into two groups, draining (DG) and non‐draining groups (NDG), was carried out using the block method. Participants were divided into 58 blocks, each comprising four individuals. We initially established six blocks representing all possible random allocations of patients into the groups, followed by the creation of the remaining blocks using an Excel sequence.

### 2.2 Preoperation details

All patients were admitted on the day or the day before of the operation and prepared for elective surgery, and in the hands of consultants and senior year residents. Some doctors prescribed antibiotics before surgery, and some did not. We collected the main characteristics of the patients, such as age, sex, body mass index, smoking status, as well as comorbidities, and performed laboratory analyzes. Patients were also categorized into five grades based on their health status using the American Society of Anaesthesiologists physical status classification.^9^

### 2.3 Operative details and interventional description

All surgeries were performed under general anesthesia and laparoscopically by inserting four trocars and a section between 10 mm clips of the cystic duct and artery was performed. The gallbladder was always retrieved through the epigastric port.

All operative details were recorded, including indications for cholecystectomy, duration of operation, difficulty of surgery using Nassar's criteria, perioperative medications, type of cholecystitis if it was inflamed, amount of drainage in the DG, type of fluid, and duration of the drain placement.

Drain was placed postoperatively and before wound closure for 24 h to 4 days. The drain type is Nelaton catheter (FR 18; RED), polyvinyl chloride plastic.

### 2.4 Postoperative and follow‐up details

All patients were followed up for 30 days from the operation day, either by phone call or hospital visit, and recording any complications such as wound infection, abscess formation, hemorrhage, pancreatitis, and postoperative prolonged shoulder pain. Complications were graded based on the Clavien−Dindo classification.^10^ Furthermore, the complication management and additional medications prescribed for the complication were recorded.

### 2.5 Statistical analysis

We conducted an intention‐to‐treat analysis. Quantitative data were analyzed using an independent *t*‐test to compare between the two groups, while qualitative data were analyzed using a chi‐square test. All tests were two‐tailed, and the level of significance was set at 0.05. Data compilation was done by an independent participant who was unaware of patients' allocation, and the results were analyzed using the SPSS PC version 24.0 statistical software.

## 3 RESULTS

### 3.1 Participants and recruitment

This study included 232 patients who underwent laparoscopic cholecystectomy in the General Surgery Unit at Aleppo University Hospital, during the period from February 1 to November 30, 2022.

**Figure 1:**
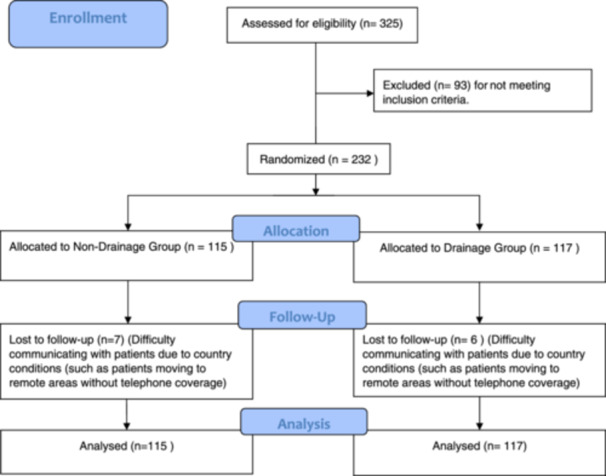
Profile of the trial.

The patients were randomly divided into two groups, where group DG (*n* = 117) was the group for whom the drainage was placed, and group NDG (*n* = 115) was the one who did not have the drainage. Most of the sample was females (*n* = 196; 84.48%). We lost the follow‐up for 6/117 (5.1%) from the DG and 7/115 from NDG (5.9%) (Figure 1).

### 3.2 Main characteristics and laboratory findings of the patients

There was no statistical difference between the main characteristics between the two groups, which included age (mean DG = 45.1, SD = 1.4 vs. mean NDG = 41.4, SD = 1.3), gender (male DG = 22 [18.8%] vs. male NDG = 14 [12.1%], *p* = 0.163), ASA score, and BMI value, indication for cholecystectomy, and smoking status, and they are detailed and compared in Table 1.

**Table 1:** Main characteristics of the patients.

Characteristic		Drainage group	Non‐drainage group	Total	*p* Value
Abdominal drainage		117	115	232	
Gender—male (*n*, %)		22 (18.8%)	14 (12.1%)	36 (15.5%)	0.163
Age (years) mean (SD)		45.10 (14.66)	41.39 (13.84)		0.483
ASA					0.647
	Class I (*n*, %)	(60, 51.28%)	(66, 57.39%)	126	
	Class II (*n*, %)	(50, 42.73%)	(43, 37.39%)	93	
	Class III (*n*, %)	(7, 5.9%)	(6, 5.2%)	13	
	Class IV (*n*, %)	0, 0	0, 0	0	
	Class V (*n*, %)	0, 0	0, 0	0	
BMI mean (SD)		28.93 (5.98)	28.54 (6.05)		0.900
Indication for cholecystectomy (*n*, %)					
	Biliary colic	107 (91.45%)	102 (88.69%)	209	0.482
	Choledocholithiasis	0	1 (0.89%)	1	0.312
	Cholangitis	2 (1.7%)	3 (2.6%)	5	0.115
	Biliary pancreatitis	1 (0.85%)	1 (0.89%)	2	0.990
	Cholecystitis	7 (6.0%)	9 (7.8%)	15	0.320
Smoking					
	A daily smoker (*n*, %)	(24, 20.5%)	(19, 16.5%)	43	0.122
	An occasional smoker (*n*, %)	(2, 1.7%)	(8, 6.9%)	10	0.133
	Ex‐smoker (*n*, %)	(4, 3.4%)	(2, 1.7%)	6	0.420
	Nonsmoker (*n*, %)	(86, 73.5%)	(87, 75.6%)	173	0.941

Abbreviation: BMI, body mass index.

ASA I: Healthy person.

ASA II: Mild systemic disease.

ASA III: Severe systemic disease.

ASA IV: Severe systemic disease that is a constant threat to life.

ASA IV: A moribund person who is not expected to survive without the operation.

ASA V: A declared brain‐dead person whose organs are being removed for donor purposes.

Moreover, there was no statistical difference between the comorbidities and laboratory findings of the patients. The values of data, laboratory findings and comorbidities of the patients, and perioperative details were analyzed and their results organized in Table 2.

**Table 2:** Comorbidities and laboratory findings of the patients.

Characteristic		Drainage group	Non‐drainage group	Total	*p* Value
Comorbidities (*n*/*N*)					
	Previous open abdominal	31	36	67	0.419
	Diabetes mellitus	9	4	13	0.163
	Hypertension requiring medication	24	21	45	0.664
	Ischemic heart disease	2	2	4	0.986
	Chronic obstructive pulmonary disease	1	3	4	0.364
	Urinary tract infection	14	7	21	0.119
	Chronic kidney disease	0	0	0	
	Known liver cirrhosis	0	1	1	0.312
	Past history of COVID‐19 infection (within the last 12 months)	2	5	7	0.240
	Cerebrovascular accident	0	1	1	0.312
	Deep vein thrombosis	0	0	0	
	Asthma	1	6	7	0.052
	Chronic immunosuppression	1	0	1	0.320
	Other	5	7	12	0.533
Laboratory findings mean (SD)/*N*					
	Hemoglobin (g/dL)	12.24 (1.50)/115	12.24 (1.28)/115		0.983
	WBC 10^9^/L	7.75 (2.34)/115	7.46 (2.13)/114		0.342
	Platelet (10^3^/μL)	265.77 (83.3)/111	257.76 (75.1)/112		0.452
	Bilirubin total (mg/dL)	0.6994 (0.52)/106	0.6381 (0.28)/93		0.315
	Bilirubin direct (mg/dL)	0.33 (0.84)/98	0.23 (0.17)/84		0.307
	Alkaline phosphatase	95.432 (37.5)/98	90.5 (43.1)/93		0.407
	AST (U/L)	28.1 (21.5)/101	26.3 (13.4)/106		0.462
	ALT (U/L)	24 (13)/102	24 (13)/103		0.978
	Glucose (mg/dL)	102.42 (37)/98	101 (34)/99		0.881

### 3.3 Perioperative outcomes

Thirty‐one patients (13.3%) had cholecystitis, 20 of whom were in DG and 11 patients in NDG. The vast majority of these cases (*n* = 29) were mild cholecystitis (Grade l). The duration of the surgical operation (including placing the drain) was the most important parameter for comparison, as the mean duration, in minutes, in NDG (mean = 44.92, SD = 1.85) was shorter than that in DG (mean 55.14, SD = 2.14), with (*p* = 0.039) statistically significant, which this indicates that the use of the drainage led to a prolongation of the surgical operation.

There was no statistical difference between the two groups in terms of the use of prophylactic antibiotics, the difficulty of surgical work according to Nassar's grade, in addition to the use of medications preoperatively as shown in Table 3.

**Table 3:** Perioperative details.

Characteristic		Drainage group	Non‐drainage group	Total	*p* Value
Prophylactic antibiotic (*n*/*N*)		22/117	19/115	41/232	0.649
Operation duration (in min) (median, range)/*N*		55.14 (22.88)/117	44.92 (19.62)/115		0.039
Postoperative hospital stays, days (median, range)		1 [1−4]	1		
Nassar grade (*n*)					
	N1	55	67	122	0.121
	N2	44	37	81	0.377
	N3	10	8	18	0.546
	N4	8	3	11	0.195
Perioperative medication (*n*/*N*)					
	NSAIDs	80	72	152	0.325
	Paracetamol	111	110	221	0.568
	Opioids	11	15	26	0.432
	PPI	49	43	92	0.441
	Penicillin	1	4	5	0.244
	Cephalosporins	17	10	27	0.219
	Fluoroquinolones	1	0	1	0.370
	Metronidazol	4	2	6	0.652
	Ondansetron	52	52	104	0.612
Any cholecystitis (*n*/*N*)					
	Acute noncomplicated	18	11	29	0.278
	Pericholecystic collection	0	0	0	
	Mucocele	2	0	2	0.278
	Empyema	0	0	0	

The type and quantity of the output from the drain in DG was determined by the surgical team. Where was as the follows: serosanguineous *n* = 70 (59.8%), clear *n* = 38 (32.5%), and bloody *n* = 8 (6.8%) as shown in Table 4.

**Table 4:** Drainage outcomes.

Output of drainage	Drainage group results (117)	
Amount of drainage (mL, median, range)		5 (0−200)
Type of the output (*n*, %)		
	Serosanguineous	70 (59.8%)
	Clear	38 (32.5%)
	Pus	0
	Bloody	8 (6.8%)
	Bile	1 (0.8%)
Length of using a drainage (in days) (median, range)		1 (0−4)

### 3.4 30 days follow‐up outcomes

The follow‐up period of surgical outcomes for all patients continued for 30 days, starting from the day of surgery, with the exception of some patients who could not be followed up due to difficulties in contacting them or moving to remote residential areas. And whose number was in NDG (*n* = 7), and it was (*n* = 6) in group DG. After analyzing these results, we concluded the following: The total number of complicated cases reached 22 (9.48%) cases. These previous cases were distributed as follows (DG = 9 vs. NDG = 13) with *p* = 0.348.

The observed complications can be outlined as follows: bleeding (*n* = 1) (DG = 1 vs. NDG = 0; *p* = 0.320), bile leak with no established bile duct injury (*n* = 1) (DG = 1 vs. NDG = 0; *p* = 0.320), wound infection (*n* = 12) (DG = 4 vs. NDG = 8; *p* = 0.144), urinary tract infection (*n* = 3) (DG = 0 vs. NDG = 3; *p* = 0.079), prolonged shoulder pain (*n* = 2) (DG = 0 vs. NDG = 2; *p* = 0.152), acute pancreatitis (*n* = 1) (DG = 1 vs. NDG = 0; *p* = 0.320), with four cases needed additional pharmacological treatment. No statistically significant differences were observed in the incidence of any of the aforementioned complications.

No instances of intra‐abdominal abscess, bowel injury, bile duct injury, enterocutaneous fistula, venous thromboembolism, or cardiac, respiratory, or renal complications were reported in either group. Additionally, no deaths occurred during the follow‐up period. The findings of this analysis are detailed in Table 5.

**Table 5:** 30 days follow‐up outcomes.

Outcome		Drainage group	Non‐drainage group	Total	OR and CI	*p* Value
Total complication (*n*/*N*)		9/117	13/115	22/232	0.654 (0.26−1.595)	0.348
	Wound infection	4	8	12	0.417 (0.125−1.394)	0.144
	Bleeding	1	0	1	‐	0.320
	Bile leak (minor) with no established bile duct injury and resolution without intervention	1	0	1	‐	0.320
	Intra‐abdominal abscess	0	0	0	‐	‐
	Acute pancreatitis	1	0	1	‐	0.320
	Urinary tract infection (UTI)	0	3	3	‐	0.079
	Prolonged postoperative shoulder pain	0	2	2	‐	0.152
	Other	0	0	0	1.487 (0.244−9.067)	0.665
Clavien−Dindo classification (*n*/*N*)						
	Grade I	6	10	16	0.568 (0.199−1.617)	0.284
	Grade II	1	4	5	0.239 (0.026−2.174)	0.169
	Grade III	2	0	2	‐	0.159
	Grade IV	0	0	0	‐	‐
	Grade V	0	0	0	‐	‐

Abbreviation: OR and CI, odds ratio and confidence interval (lower, upper).

## 4 DISCUSSION

The study included 232 patients, predominantly female, who were randomly assigned to either the DG or NDG. There were no significant differences in main characteristics between the groups, suggesting a well‐matched baseline. Loss to follow‐up rates were low in both groups, minimizing potential bias. Analysis showed no significant discrepancies in comorbidities and lab findings, enhancing internal validity. The homogeneity in patient characteristics and findings between groups supports attributing outcome differences to drainage during cholecystectomy.

In our investigation, we observed a notable distinction in the surgery duration between the DG and the NDG. The average surgery time, which involved drain placement, was markedly lengthier in the DG at 55.14 min compared to the NDG at 44.92 min, with a *p*‐value of 0.039, signifying statistical significance. Interestingly, despite this variance in operation duration, the postoperative hospital stay remained consistent among both groups, with a median of 1 day for each. This aligns with a separate randomized, prospective study on 300 cholecystectomies that further explored the efficacy of drainage, where no discernible difference in hospital stay length was noted.^11^

The outcomes of our trial exhibit wound infection as the most frequent complication in both groups (12 out of 232 cases, 5.2%), although this finding did not reach statistical significance. This is in line with the conclusions of prior systematic reviews.^12^ The same trend is observed for other complications like bleeding, bile leakage, prolonged shoulder discomfort, and acute pancreatitis. While these findings have significantly reduced the routine use of drainage, they have not done so permanently, leaving this practice subject to ongoing debate and controversy. Our previous umbrella review on the efficacy and safety of prophylactic drainage post‐intra‐abdominal surgeries also failed to demonstrate any advantages from routine drainage application.^13^

The significance of conducting subgroup analyses, considering factors like health status, medical history, and habits like smoking, is paramount in tailoring patient care to individual needs. However, our findings revealed that the relevance of drain placement about wound infection and Cleven−Dindo classification did not vary between the two groups across different subgroups such as smoking status, diabetic patients, and those with a health status classified as ASA II−III (Supporting Information S1: File A) To our knowledge, no previous studies compare the use of prophylactic drainage or not after elective cholecystectomy in these groups of patients.

There are some limitations to our study, as most of our sample were females (cholecystectomy is more common in females than men). Our study did not study the financial aspect, as the hospital is academic, and all patients were provided with free care. This lack of financial analysis may limit the transferability of our findings to healthcare systems with different payment structures or resource constraints. Furthermore, our study focused on one specific type of drain, potentially overlooking the nuances and potential variations in outcomes associated with different drain models. We did not calculate sample size, but rather, we relied on including all qualified patients based on the study criteria within 9 months and one center.

## 5 CONCLUSION

The findings of our study demonstrate a well‐designed, internally valid investigation into the impact of drainage during cholecystectomy. The significant difference in surgery duration between the DG and NDG sheds light on the potential influence of drain placement on operation times. While wound infection emerged as the most common complication, its occurrence did not significantly differ between the groups, aligning with previous research. The lack of variation in outcomes across different patient subgroups suggests that the relevance of drain placement remains insignificant. Accordingly, the use of prophylactic drainage was safe, but ineffective, as it did not improve the outcomes statistically significantly or worsen them, which is consistent with previous studies. These insights contribute to the ongoing debate surrounding the routine use of prophylactic drainage post‐cholecystectomy, highlighting the need for personalized patient care in this setting.

## AUTHOR CONTRIBUTIONS

Ahmad Y. Arnaout coordinated the study, developed the methodology, validated the findings, conducted data analysis, interpreted the data, contributed to the original manuscript draft, and reviewed the final version. Lama Kadoura and Ruqaya Masri coordinated the study, developed the methodology, validated the findings, and reviewed the final version of the manuscript. Yaman Nerabani and Abd Alazeez Atli coordinated the study, validated the findings, contributed to the original manuscript draft, and reviewed the final version. Khaled Arnaout conducted data analysis and interpreted the data. Ibrahim Arnaout contributed to the original draft of the manuscript. Adel Bsata, Hasan Kayali, Nehad Mahli, Ahmad Al‐Haj, Kusay Ayoub, and Ammar Niazi served as scientific supervisors and study coordinators. Ahmad Ghazal served as scientific supervisor, and study coordinator, interpreted the data, validated the findings, and critically reviewed the manuscript. The Aleppo University Hospital Team played a critical role in data collection for this study.

## CONFLICT OF INTEREST STATEMENT

The authors declare no conflict of interest.

## ETHICS STATEMENT

This study was performed in accordance with the ethical standards as laid down in the 1964 Declaration of Helsinki and its later amendments, and under ethical approval from the ethics committee at the Faculty of Medicine, University of Aleppo, Syria.

## TRANSPARENCY STATEMENT

The lead author, Ahmad Y. Arnaout, affirms that this manuscript is an honest, accurate, and transparent account of the study being reported, that no important aspects of the study have been omitted, and that any discrepancies from the study as planned (and, if relevant, registered) have been explained.

## Supporting information

Supporting information.

## Data Availability

The data that support the findings of this study are available on request from the corresponding author. The data are not publicly available due to privacy.
