# Supplementary material for: The efficacy and safety of using prophylactic abdominal drainage after laparoscopic cholecystectomy: A randomized control trial
Source: Health Sci Rep. 2024 Aug 8;7(8):e2284. doi: 10.1002/hsr2.2284 (PMC11310261; doi:10.1002/hsr2.2284)
Supplement: Supplementary file 1 — Supporting information. [file HSR2-7-e2284-s001.docx]

**Supplementary File A: Subgroups analysis**

| **Case Processing Summary** | | | | | | |
| --- | --- | --- | --- | --- | --- | --- |
|  | Cases | | | | | |
|  | Valid | | Missing | | Total | |
|  | N | Percent | N | Percent | N | Percent |
| Abdominal drain * Clavien-Dindo classification Grade I * ASA class | 232 | 99.6% | 1 | 0.4% | 233 | 100.0% |
| Abdominal drain * Clavien-Dindo classification Grade I * Diabetes mellitus | 232 | 99.6% | 1 | 0.4% | 233 | 100.0% |
| Abdominal drain * Clavien-Dindo classification Grade I * Active smoker (Yes/No) | 232 | 99.6% | 1 | 0.4% | 233 | 100.0% |
| Abdominal drain * Clavien-Dindo classification Grade I * Grade I (mild) | 232 | 99.6% | 1 | 0.4% | 233 | 100.0% |
| Abdominal drain * Clavien-Dindo classification Grade I * Grade II (moderate) | 232 | 99.6% | 1 | 0.4% | 233 | 100.0% |
| Abdominal drain * Clavien-Dindo classification Grade I * Grade III (severe) | 31 | 13.3% | 202 | 86.7% | 233 | 100.0% |
| Abdominal drain * Grade II * ASA class | 232 | 99.6% | 1 | 0.4% | 233 | 100.0% |
| Abdominal drain * Grade II * Diabetes mellitus | 232 | 99.6% | 1 | 0.4% | 233 | 100.0% |
| Abdominal drain * Grade II * Active smoker (Yes/No) | 232 | 99.6% | 1 | 0.4% | 233 | 100.0% |
| Abdominal drain * Grade II * Grade I (mild) | 232 | 99.6% | 1 | 0.4% | 233 | 100.0% |
| Abdominal drain * Grade II * Grade II (moderate) | 232 | 99.6% | 1 | 0.4% | 233 | 100.0% |
| Abdominal drain * Grade II * Grade III (severe) | 31 | 13.3% | 202 | 86.7% | 233 | 100.0% |
| Abdominal drain * Grade III * ASA class | 232 | 99.6% | 1 | 0.4% | 233 | 100.0% |
| Abdominal drain * Grade III * Diabetes mellitus | 232 | 99.6% | 1 | 0.4% | 233 | 100.0% |
| Abdominal drain * Grade III * Active smoker (Yes/No) | 232 | 99.6% | 1 | 0.4% | 233 | 100.0% |
| Abdominal drain * Grade III * Grade I (mild) | 232 | 99.6% | 1 | 0.4% | 233 | 100.0% |
| Abdominal drain * Grade III * Grade II (moderate) | 232 | 99.6% | 1 | 0.4% | 233 | 100.0% |
| Abdominal drain * Grade III * Grade III (severe) | 31 | 13.3% | 202 | 86.7% | 233 | 100.0% |
| Abdominal drain * Wound infection * ASA class | 232 | 99.6% | 1 | 0.4% | 233 | 100.0% |
| Abdominal drain * Wound infection * Diabetes mellitus | 232 | 99.6% | 1 | 0.4% | 233 | 100.0% |
| Abdominal drain * Wound infection * Active smoker (Yes/No) | 232 | 99.6% | 1 | 0.4% | 233 | 100.0% |
| Abdominal drain * Wound infection * Grade I (mild) | 232 | 99.6% | 1 | 0.4% | 233 | 100.0% |
| Abdominal drain * Wound infection * Grade II (moderate) | 232 | 99.6% | 1 | 0.4% | 233 | 100.0% |
| Abdominal drain * Wound infection * Grade III (severe) | 31 | 13.3% | 202 | 86.7% | 233 | 100.0% |

**Abdominal drain * Clavien-Dindo classification Grade I * ASA class**

| **Crosstab** | | | | | | |
| --- | --- | --- | --- | --- | --- | --- |
| ASA class | | | | Clavien-Dindo classification Grade I | | Total |
|  |  |  |  | 0 | 1 |  |
| 1 | Abdominal drain | 0 | Count | 61 | 4 | 65 |
|  |  |  | % within Abdominal drain | 93.8% | 6.2% | 100.0% |
|  |  |  | % within Clavien-Dindo classification Grade I | 51.7% | 57.1% | 52.0% |
|  |  |  | % of Total | 48.8% | 3.2% | 52.0% |
|  |  | 1 | Count | 57 | 3 | 60 |
|  |  |  | % within Abdominal drain | 95.0% | 5.0% | 100.0% |
|  |  |  | % within Clavien-Dindo classification Grade I | 48.3% | 42.9% | 48.0% |
|  |  |  | % of Total | 45.6% | 2.4% | 48.0% |
|  | Total | | Count | 118 | 7 | 125 |
|  |  |  | % within Abdominal drain | 94.4% | 5.6% | 100.0% |
|  |  |  | % within Clavien-Dindo classification Grade I | 100.0% | 100.0% | 100.0% |
|  |  |  | % of Total | 94.4% | 5.6% | 100.0% |
| 2 | Abdominal drain | 0 | Count | 39 | 5 | 44 |
|  |  |  | % within Abdominal drain | 88.6% | 11.4% | 100.0% |
|  |  |  | % within Clavien-Dindo classification Grade I | 45.3% | 62.5% | 46.8% |
|  |  |  | % of Total | 41.5% | 5.3% | 46.8% |
|  |  | 1 | Count | 47 | 3 | 50 |
|  |  |  | % within Abdominal drain | 94.0% | 6.0% | 100.0% |
|  |  |  | % within Clavien-Dindo classification Grade I | 54.7% | 37.5% | 53.2% |
|  |  |  | % of Total | 50.0% | 3.2% | 53.2% |
|  | Total | | Count | 86 | 8 | 94 |
|  |  |  | % within Abdominal drain | 91.5% | 8.5% | 100.0% |
|  |  |  | % within Clavien-Dindo classification Grade I | 100.0% | 100.0% | 100.0% |
|  |  |  | % of Total | 91.5% | 8.5% | 100.0% |
| 3 | Abdominal drain | 0 | Count | 5 | 1 | 6 |
|  |  |  | % within Abdominal drain | 83.3% | 16.7% | 100.0% |
|  |  |  | % within Clavien-Dindo classification Grade I | 41.7% | 100.0% | 46.2% |
|  |  |  | % of Total | 38.5% | 7.7% | 46.2% |
|  |  | 1 | Count | 7 | 0 | 7 |
|  |  |  | % within Abdominal drain | 100.0% | 0.0% | 100.0% |
|  |  |  | % within Clavien-Dindo classification Grade I | 58.3% | 0.0% | 53.8% |
|  |  |  | % of Total | 53.8% | 0.0% | 53.8% |
|  | Total | | Count | 12 | 1 | 13 |
|  |  |  | % within Abdominal drain | 92.3% | 7.7% | 100.0% |
|  |  |  | % within Clavien-Dindo classification Grade I | 100.0% | 100.0% | 100.0% |
|  |  |  | % of Total | 92.3% | 7.7% | 100.0% |
| Total | Abdominal drain | 0 | Count | 105 | 10 | 115 |
|  |  |  | % within Abdominal drain | 91.3% | 8.7% | 100.0% |
|  |  |  | % within Clavien-Dindo classification Grade I | 48.6% | 62.5% | 49.6% |
|  |  |  | % of Total | 45.3% | 4.3% | 49.6% |
|  |  | 1 | Count | 111 | 6 | 117 |
|  |  |  | % within Abdominal drain | 94.9% | 5.1% | 100.0% |
|  |  |  | % within Clavien-Dindo classification Grade I | 51.4% | 37.5% | 50.4% |
|  |  |  | % of Total | 47.8% | 2.6% | 50.4% |
|  | Total | | Count | 216 | 16 | 232 |
|  |  |  | % within Abdominal drain | 93.1% | 6.9% | 100.0% |
|  |  |  | % within Clavien-Dindo classification Grade I | 100.0% | 100.0% | 100.0% |
|  |  |  | % of Total | 93.1% | 6.9% | 100.0% |

| **Chi-Square Tests** | | | | | | |
| --- | --- | --- | --- | --- | --- | --- |
| ASA class | | Value | df | Asymptotic Significance (2-sided) | Exact Sig. (2-sided) | Exact Sig. (1-sided) |
| 1 | Pearson Chi-Square | .079^c^ | 1 | .779 |  |  |
|  | Continuity Correction^b^ | .000 | 1 | 1.000 |  |  |
|  | Likelihood Ratio | .079 | 1 | .779 |  |  |
|  | Fisher's Exact Test |  |  |  | 1.000 | .545 |
|  | Linear-by-Linear Association | .078 | 1 | .780 |  |  |
|  | N of Valid Cases | 125 |  |  |  |  |
| 2 | Pearson Chi-Square | .865^d^ | 1 | .352 |  |  |
|  | Continuity Correction^b^ | .313 | 1 | .576 |  |  |
|  | Likelihood Ratio | .867 | 1 | .352 |  |  |
|  | Fisher's Exact Test |  |  |  | .467 | .288 |
|  | Linear-by-Linear Association | .856 | 1 | .355 |  |  |
|  | N of Valid Cases | 94 |  |  |  |  |
| 3 | Pearson Chi-Square | 1.264^e^ | 1 | .261 |  |  |
|  | Continuity Correction^b^ | .006 | 1 | .936 |  |  |
|  | Likelihood Ratio | 1.644 | 1 | .200 |  |  |
|  | Fisher's Exact Test |  |  |  | .462 | .462 |
|  | Linear-by-Linear Association | 1.167 | 1 | .280 |  |  |
|  | N of Valid Cases | 13 |  |  |  |  |
| Total | Pearson Chi-Square | 1.150^a^ | 1 | .284 |  |  |
|  | Continuity Correction^b^ | .661 | 1 | .416 |  |  |
|  | Likelihood Ratio | 1.160 | 1 | .281 |  |  |
|  | Fisher's Exact Test |  |  |  | .312 | .208 |
|  | Linear-by-Linear Association | 1.145 | 1 | .285 |  |  |
|  | N of Valid Cases | 232 |  |  |  |  |
| a. 0 cells (0.0%) have expected count less than 5. The minimum expected count is 7.93. | | | | | | |
| b. Computed only for a 2x2 table | | | | | | |
| c. 2 cells (50.0%) have expected count less than 5. The minimum expected count is 3.36. | | | | | | |
| d. 2 cells (50.0%) have expected count less than 5. The minimum expected count is 3.74. | | | | | | |
| e. 2 cells (50.0%) have expected count less than 5. The minimum expected count is .46. | | | | | | |

**Abdominal drain * Clavien-Dindo classification Grade I * Diabetes mellitus**

| **Crosstab** | | | | | | |
| --- | --- | --- | --- | --- | --- | --- |
| Diabetes mellitus | | | | Clavien-Dindo classification Grade I | | Total |
|  |  |  |  | 0 | 1 |  |
| 0 | Abdominal drain | 0 | Count | 101 | 10 | 111 |
|  |  |  | % within Abdominal drain | 91.0% | 9.0% | 100.0% |
|  |  |  | % within Clavien-Dindo classification Grade I | 49.8% | 62.5% | 50.7% |
|  |  |  | % of Total | 46.1% | 4.6% | 50.7% |
|  |  | 1 | Count | 102 | 6 | 108 |
|  |  |  | % within Abdominal drain | 94.4% | 5.6% | 100.0% |
|  |  |  | % within Clavien-Dindo classification Grade I | 50.2% | 37.5% | 49.3% |
|  |  |  | % of Total | 46.6% | 2.7% | 49.3% |
|  | Total | | Count | 203 | 16 | 219 |
|  |  |  | % within Abdominal drain | 92.7% | 7.3% | 100.0% |
|  |  |  | % within Clavien-Dindo classification Grade I | 100.0% | 100.0% | 100.0% |
|  |  |  | % of Total | 92.7% | 7.3% | 100.0% |
| 1 | Abdominal drain | 0 | Count | 4 |  | 4 |
|  |  |  | % within Abdominal drain | 100.0% |  | 100.0% |
|  |  |  | % within Clavien-Dindo classification Grade I | 30.8% |  | 30.8% |
|  |  |  | % of Total | 30.8% |  | 30.8% |
|  |  | 1 | Count | 9 |  | 9 |
|  |  |  | % within Abdominal drain | 100.0% |  | 100.0% |
|  |  |  | % within Clavien-Dindo classification Grade I | 69.2% |  | 69.2% |
|  |  |  | % of Total | 69.2% |  | 69.2% |
|  | Total | | Count | 13 |  | 13 |
|  |  |  | % within Abdominal drain | 100.0% |  | 100.0% |
|  |  |  | % within Clavien-Dindo classification Grade I | 100.0% |  | 100.0% |
|  |  |  | % of Total | 100.0% |  | 100.0% |
| Total | Abdominal drain | 0 | Count | 105 | 10 | 115 |
|  |  |  | % within Abdominal drain | 91.3% | 8.7% | 100.0% |
|  |  |  | % within Clavien-Dindo classification Grade I | 48.6% | 62.5% | 49.6% |
|  |  |  | % of Total | 45.3% | 4.3% | 49.6% |
|  |  | 1 | Count | 111 | 6 | 117 |
|  |  |  | % within Abdominal drain | 94.9% | 5.1% | 100.0% |
|  |  |  | % within Clavien-Dindo classification Grade I | 51.4% | 37.5% | 50.4% |
|  |  |  | % of Total | 47.8% | 2.6% | 50.4% |
|  | Total | | Count | 216 | 16 | 232 |
|  |  |  | % within Abdominal drain | 93.1% | 6.9% | 100.0% |
|  |  |  | % within Clavien-Dindo classification Grade I | 100.0% | 100.0% | 100.0% |
|  |  |  | % of Total | 93.1% | 6.9% | 100.0% |

| **Chi-Square Tests** | | | | | | |
| --- | --- | --- | --- | --- | --- | --- |
| Diabetes mellitus | | Value | df | Asymptotic Significance (2-sided) | Exact Sig. (2-sided) | Exact Sig. (1-sided) |
| 0 | Pearson Chi-Square | .964^c^ | 1 | .326 |  |  |
|  | Continuity Correction^b^ | .522 | 1 | .470 |  |  |
|  | Likelihood Ratio | .975 | 1 | .324 |  |  |
|  | Fisher's Exact Test |  |  |  | .438 | .236 |
|  | Linear-by-Linear Association | .960 | 1 | .327 |  |  |
|  | N of Valid Cases | 219 |  |  |  |  |
| 1 | Pearson Chi-Square | .^d^ |  |  |  |  |
|  | N of Valid Cases | 13 |  |  |  |  |
| Total | Pearson Chi-Square | 1.150^a^ | 1 | .284 |  |  |
|  | Continuity Correction^b^ | .661 | 1 | .416 |  |  |
|  | Likelihood Ratio | 1.160 | 1 | .281 |  |  |
|  | Fisher's Exact Test |  |  |  | .312 | .208 |
|  | Linear-by-Linear Association | 1.145 | 1 | .285 |  |  |
|  | N of Valid Cases | 232 |  |  |  |  |
| a. 0 cells (0.0%) have expected count less than 5. The minimum expected count is 7.93. | | | | | | |
| b. Computed only for a 2x2 table | | | | | | |
| c. 0 cells (0.0%) have expected count less than 5. The minimum expected count is 7.89. | | | | | | |
| d. No statistics are computed because Clavien-Dindo classification Grade I is a constant. | | | | | | |

**Abdominal drain * Clavien-Dindo classification Grade I * Active smoker (Yes/No)**

| **Crosstab** | | | | | | |
| --- | --- | --- | --- | --- | --- | --- |
| Active smoker (Yes/No) | | | | Clavien-Dindo classification Grade I | | Total |
|  |  |  |  | 0 | 1 |  |
| 0 | Abdominal drain | 0 | Count | 79 | 9 | 88 |
|  |  |  | % within Abdominal drain | 89.8% | 10.2% | 100.0% |
|  |  |  | % within Clavien-Dindo classification Grade I | 47.9% | 64.3% | 49.2% |
|  |  |  | % of Total | 44.1% | 5.0% | 49.2% |
|  |  | 1 | Count | 86 | 5 | 91 |
|  |  |  | % within Abdominal drain | 94.5% | 5.5% | 100.0% |
|  |  |  | % within Clavien-Dindo classification Grade I | 52.1% | 35.7% | 50.8% |
|  |  |  | % of Total | 48.0% | 2.8% | 50.8% |
|  | Total | | Count | 165 | 14 | 179 |
|  |  |  | % within Abdominal drain | 92.2% | 7.8% | 100.0% |
|  |  |  | % within Clavien-Dindo classification Grade I | 100.0% | 100.0% | 100.0% |
|  |  |  | % of Total | 92.2% | 7.8% | 100.0% |
| 1 | Abdominal drain | 0 | Count | 26 | 1 | 27 |
|  |  |  | % within Abdominal drain | 96.3% | 3.7% | 100.0% |
|  |  |  | % within Clavien-Dindo classification Grade I | 51.0% | 50.0% | 50.9% |
|  |  |  | % of Total | 49.1% | 1.9% | 50.9% |
|  |  | 1 | Count | 25 | 1 | 26 |
|  |  |  | % within Abdominal drain | 96.2% | 3.8% | 100.0% |
|  |  |  | % within Clavien-Dindo classification Grade I | 49.0% | 50.0% | 49.1% |
|  |  |  | % of Total | 47.2% | 1.9% | 49.1% |
|  | Total | | Count | 51 | 2 | 53 |
|  |  |  | % within Abdominal drain | 96.2% | 3.8% | 100.0% |
|  |  |  | % within Clavien-Dindo classification Grade I | 100.0% | 100.0% | 100.0% |
|  |  |  | % of Total | 96.2% | 3.8% | 100.0% |
| Total | Abdominal drain | 0 | Count | 105 | 10 | 115 |
|  |  |  | % within Abdominal drain | 91.3% | 8.7% | 100.0% |
|  |  |  | % within Clavien-Dindo classification Grade I | 48.6% | 62.5% | 49.6% |
|  |  |  | % of Total | 45.3% | 4.3% | 49.6% |
|  |  | 1 | Count | 111 | 6 | 117 |
|  |  |  | % within Abdominal drain | 94.9% | 5.1% | 100.0% |
|  |  |  | % within Clavien-Dindo classification Grade I | 51.4% | 37.5% | 50.4% |
|  |  |  | % of Total | 47.8% | 2.6% | 50.4% |
|  | Total | | Count | 216 | 16 | 232 |
|  |  |  | % within Abdominal drain | 93.1% | 6.9% | 100.0% |
|  |  |  | % within Clavien-Dindo classification Grade I | 100.0% | 100.0% | 100.0% |
|  |  |  | % of Total | 93.1% | 6.9% | 100.0% |

| **Chi-Square Tests** | | | | | | |
| --- | --- | --- | --- | --- | --- | --- |
| Active smoker (Yes/No) | | Value | df | Asymptotic Significance (2-sided) | Exact Sig. (2-sided) | Exact Sig. (1-sided) |
| 0 | Pearson Chi-Square | 1.390^c^ | 1 | .238 |  |  |
|  | Continuity Correction^b^ | .811 | 1 | .368 |  |  |
|  | Likelihood Ratio | 1.406 | 1 | .236 |  |  |
|  | Fisher's Exact Test |  |  |  | .276 | .184 |
|  | Linear-by-Linear Association | 1.382 | 1 | .240 |  |  |
|  | N of Valid Cases | 179 |  |  |  |  |
| 1 | Pearson Chi-Square | .001^d^ | 1 | .978 |  |  |
|  | Continuity Correction^b^ | .000 | 1 | 1.000 |  |  |
|  | Likelihood Ratio | .001 | 1 | .978 |  |  |
|  | Fisher's Exact Test |  |  |  | 1.000 | .745 |
|  | Linear-by-Linear Association | .001 | 1 | .979 |  |  |
|  | N of Valid Cases | 53 |  |  |  |  |
| Total | Pearson Chi-Square | 1.150^a^ | 1 | .284 |  |  |
|  | Continuity Correction^b^ | .661 | 1 | .416 |  |  |
|  | Likelihood Ratio | 1.160 | 1 | .281 |  |  |
|  | Fisher's Exact Test |  |  |  | .312 | .208 |
|  | Linear-by-Linear Association | 1.145 | 1 | .285 |  |  |
|  | N of Valid Cases | 232 |  |  |  |  |
| a. 0 cells (0.0%) have expected count less than 5. The minimum expected count is 7.93. | | | | | | |
| b. Computed only for a 2x2 table | | | | | | |
| c. 0 cells (0.0%) have expected count less than 5. The minimum expected count is 6.88. | | | | | | |
| d. 2 cells (50.0%) have expected count less than 5. The minimum expected count is .98. | | | | | | |

**Abdominal drain * Clavien-Dindo classification Grade I * Grade I (mild)**

| **Crosstab** | | | | | | |
| --- | --- | --- | --- | --- | --- | --- |
| Grade I (mild) | | | | Clavien-Dindo classification Grade I | | Total |
|  |  |  |  | 0 | 1 |  |
|  | Abdominal drain | 0 | Count | 95 | 9 | 104 |
|  |  |  | % within Abdominal drain | 91.3% | 8.7% | 100.0% |
|  |  |  | % within Clavien-Dindo classification Grade I | 51.1% | 60.0% | 51.7% |
|  |  |  | % of Total | 47.3% | 4.5% | 51.7% |
|  |  | 1 | Count | 91 | 6 | 97 |
|  |  |  | % within Abdominal drain | 93.8% | 6.2% | 100.0% |
|  |  |  | % within Clavien-Dindo classification Grade I | 48.9% | 40.0% | 48.3% |
|  |  |  | % of Total | 45.3% | 3.0% | 48.3% |
|  | Total | | Count | 186 | 15 | 201 |
|  |  |  | % within Abdominal drain | 92.5% | 7.5% | 100.0% |
|  |  |  | % within Clavien-Dindo classification Grade I | 100.0% | 100.0% | 100.0% |
|  |  |  | % of Total | 92.5% | 7.5% | 100.0% |
| 0 | Abdominal drain | 1 | Count | 2 |  | 2 |
|  |  |  | % within Abdominal drain | 100.0% |  | 100.0% |
|  |  |  | % within Clavien-Dindo classification Grade I | 100.0% |  | 100.0% |
|  |  |  | % of Total | 100.0% |  | 100.0% |
|  | Total | | Count | 2 |  | 2 |
|  |  |  | % within Abdominal drain | 100.0% |  | 100.0% |
|  |  |  | % within Clavien-Dindo classification Grade I | 100.0% |  | 100.0% |
|  |  |  | % of Total | 100.0% |  | 100.0% |
| 1 | Abdominal drain | 0 | Count | 10 | 1 | 11 |
|  |  |  | % within Abdominal drain | 90.9% | 9.1% | 100.0% |
|  |  |  | % within Clavien-Dindo classification Grade I | 35.7% | 100.0% | 37.9% |
|  |  |  | % of Total | 34.5% | 3.4% | 37.9% |
|  |  | 1 | Count | 18 | 0 | 18 |
|  |  |  | % within Abdominal drain | 100.0% | 0.0% | 100.0% |
|  |  |  | % within Clavien-Dindo classification Grade I | 64.3% | 0.0% | 62.1% |
|  |  |  | % of Total | 62.1% | 0.0% | 62.1% |
|  | Total | | Count | 28 | 1 | 29 |
|  |  |  | % within Abdominal drain | 96.6% | 3.4% | 100.0% |
|  |  |  | % within Clavien-Dindo classification Grade I | 100.0% | 100.0% | 100.0% |
|  |  |  | % of Total | 96.6% | 3.4% | 100.0% |
| Total | Abdominal drain | 0 | Count | 105 | 10 | 115 |
|  |  |  | % within Abdominal drain | 91.3% | 8.7% | 100.0% |
|  |  |  | % within Clavien-Dindo classification Grade I | 48.6% | 62.5% | 49.6% |
|  |  |  | % of Total | 45.3% | 4.3% | 49.6% |
|  |  | 1 | Count | 111 | 6 | 117 |
|  |  |  | % within Abdominal drain | 94.9% | 5.1% | 100.0% |
|  |  |  | % within Clavien-Dindo classification Grade I | 51.4% | 37.5% | 50.4% |
|  |  |  | % of Total | 47.8% | 2.6% | 50.4% |
|  | Total | | Count | 216 | 16 | 232 |
|  |  |  | % within Abdominal drain | 93.1% | 6.9% | 100.0% |
|  |  |  | % within Clavien-Dindo classification Grade I | 100.0% | 100.0% | 100.0% |
|  |  |  | % of Total | 93.1% | 6.9% | 100.0% |

| **Chi-Square Tests** | | | | | | |
| --- | --- | --- | --- | --- | --- | --- |
| Grade I (mild) | | Value | df | Asymptotic Significance (2-sided) | Exact Sig. (2-sided) | Exact Sig. (1-sided) |
|  | Pearson Chi-Square | .443^c^ | 1 | .506 |  |  |
|  | Continuity Correction^b^ | .157 | 1 | .691 |  |  |
|  | Likelihood Ratio | .446 | 1 | .504 |  |  |
|  | Fisher's Exact Test |  |  |  | .597 | .347 |
|  | Linear-by-Linear Association | .441 | 1 | .507 |  |  |
|  | N of Valid Cases | 201 |  |  |  |  |
| 0 | Pearson Chi-Square | .^d^ |  |  |  |  |
|  | N of Valid Cases | 2 |  |  |  |  |
| 1 | Pearson Chi-Square | 1.695^e^ | 1 | .193 |  |  |
|  | Continuity Correction^b^ | .064 | 1 | .800 |  |  |
|  | Likelihood Ratio | 1.998 | 1 | .158 |  |  |
|  | Fisher's Exact Test |  |  |  | .379 | .379 |
|  | Linear-by-Linear Association | 1.636 | 1 | .201 |  |  |
|  | N of Valid Cases | 29 |  |  |  |  |
| Total | Pearson Chi-Square | 1.150^a^ | 1 | .284 |  |  |
|  | Continuity Correction^b^ | .661 | 1 | .416 |  |  |
|  | Likelihood Ratio | 1.160 | 1 | .281 |  |  |
|  | Fisher's Exact Test |  |  |  | .312 | .208 |
|  | Linear-by-Linear Association | 1.145 | 1 | .285 |  |  |
|  | N of Valid Cases | 232 |  |  |  |  |
| a. 0 cells (0.0%) have expected count less than 5. The minimum expected count is 7.93. | | | | | | |
| b. Computed only for a 2x2 table | | | | | | |
| c. 0 cells (0.0%) have expected count less than 5. The minimum expected count is 7.24. | | | | | | |
| d. No statistics are computed because Abdominal drain and Clavien-Dindo classification Grade I are constants. | | | | | | |
| e. 2 cells (50.0%) have expected count less than 5. The minimum expected count is .38. | | | | | | |

**Abdominal drain * Clavien-Dindo classification Grade I * Grade II (moderate)**

| **Crosstab** | | | | | | |
| --- | --- | --- | --- | --- | --- | --- |
| Grade II (moderate) | | | | Clavien-Dindo classification Grade I | | Total |
|  |  |  |  | 0 | 1 |  |
|  | Abdominal drain | 0 | Count | 95 | 9 | 104 |
|  |  |  | % within Abdominal drain | 91.3% | 8.7% | 100.0% |
|  |  |  | % within Clavien-Dindo classification Grade I | 51.1% | 60.0% | 51.7% |
|  |  |  | % of Total | 47.3% | 4.5% | 51.7% |
|  |  | 1 | Count | 91 | 6 | 97 |
|  |  |  | % within Abdominal drain | 93.8% | 6.2% | 100.0% |
|  |  |  | % within Clavien-Dindo classification Grade I | 48.9% | 40.0% | 48.3% |
|  |  |  | % of Total | 45.3% | 3.0% | 48.3% |
|  | Total | | Count | 186 | 15 | 201 |
|  |  |  | % within Abdominal drain | 92.5% | 7.5% | 100.0% |
|  |  |  | % within Clavien-Dindo classification Grade I | 100.0% | 100.0% | 100.0% |
|  |  |  | % of Total | 92.5% | 7.5% | 100.0% |
| 0 | Abdominal drain | 0 | Count | 10 | 1 | 11 |
|  |  |  | % within Abdominal drain | 90.9% | 9.1% | 100.0% |
|  |  |  | % within Clavien-Dindo classification Grade I | 35.7% | 100.0% | 37.9% |
|  |  |  | % of Total | 34.5% | 3.4% | 37.9% |
|  |  | 1 | Count | 18 | 0 | 18 |
|  |  |  | % within Abdominal drain | 100.0% | 0.0% | 100.0% |
|  |  |  | % within Clavien-Dindo classification Grade I | 64.3% | 0.0% | 62.1% |
|  |  |  | % of Total | 62.1% | 0.0% | 62.1% |
|  | Total | | Count | 28 | 1 | 29 |
|  |  |  | % within Abdominal drain | 96.6% | 3.4% | 100.0% |
|  |  |  | % within Clavien-Dindo classification Grade I | 100.0% | 100.0% | 100.0% |
|  |  |  | % of Total | 96.6% | 3.4% | 100.0% |
| 1 | Abdominal drain | 1 | Count | 2 |  | 2 |
|  |  |  | % within Abdominal drain | 100.0% |  | 100.0% |
|  |  |  | % within Clavien-Dindo classification Grade I | 100.0% |  | 100.0% |
|  |  |  | % of Total | 100.0% |  | 100.0% |
|  | Total | | Count | 2 |  | 2 |
|  |  |  | % within Abdominal drain | 100.0% |  | 100.0% |
|  |  |  | % within Clavien-Dindo classification Grade I | 100.0% |  | 100.0% |
|  |  |  | % of Total | 100.0% |  | 100.0% |
| Total | Abdominal drain | 0 | Count | 105 | 10 | 115 |
|  |  |  | % within Abdominal drain | 91.3% | 8.7% | 100.0% |
|  |  |  | % within Clavien-Dindo classification Grade I | 48.6% | 62.5% | 49.6% |
|  |  |  | % of Total | 45.3% | 4.3% | 49.6% |
|  |  | 1 | Count | 111 | 6 | 117 |
|  |  |  | % within Abdominal drain | 94.9% | 5.1% | 100.0% |
|  |  |  | % within Clavien-Dindo classification Grade I | 51.4% | 37.5% | 50.4% |
|  |  |  | % of Total | 47.8% | 2.6% | 50.4% |
|  | Total | | Count | 216 | 16 | 232 |
|  |  |  | % within Abdominal drain | 93.1% | 6.9% | 100.0% |
|  |  |  | % within Clavien-Dindo classification Grade I | 100.0% | 100.0% | 100.0% |
|  |  |  | % of Total | 93.1% | 6.9% | 100.0% |

| **Chi-Square Tests** | | | | | | |
| --- | --- | --- | --- | --- | --- | --- |
| Grade II (moderate) | | Value | df | Asymptotic Significance (2-sided) | Exact Sig. (2-sided) | Exact Sig. (1-sided) |
|  | Pearson Chi-Square | .443^c^ | 1 | .506 |  |  |
|  | Continuity Correction^b^ | .157 | 1 | .691 |  |  |
|  | Likelihood Ratio | .446 | 1 | .504 |  |  |
|  | Fisher's Exact Test |  |  |  | .597 | .347 |
|  | Linear-by-Linear Association | .441 | 1 | .507 |  |  |
|  | N of Valid Cases | 201 |  |  |  |  |
| 0 | Pearson Chi-Square | 1.695^d^ | 1 | .193 |  |  |
|  | Continuity Correction^b^ | .064 | 1 | .800 |  |  |
|  | Likelihood Ratio | 1.998 | 1 | .158 |  |  |
|  | Fisher's Exact Test |  |  |  | .379 | .379 |
|  | Linear-by-Linear Association | 1.636 | 1 | .201 |  |  |
|  | N of Valid Cases | 29 |  |  |  |  |
| 1 | Pearson Chi-Square | .^e^ |  |  |  |  |
|  | N of Valid Cases | 2 |  |  |  |  |
| Total | Pearson Chi-Square | 1.150^a^ | 1 | .284 |  |  |
|  | Continuity Correction^b^ | .661 | 1 | .416 |  |  |
|  | Likelihood Ratio | 1.160 | 1 | .281 |  |  |
|  | Fisher's Exact Test |  |  |  | .312 | .208 |
|  | Linear-by-Linear Association | 1.145 | 1 | .285 |  |  |
|  | N of Valid Cases | 232 |  |  |  |  |
| a. 0 cells (0.0%) have expected count less than 5. The minimum expected count is 7.93. | | | | | | |
| b. Computed only for a 2x2 table | | | | | | |
| c. 0 cells (0.0%) have expected count less than 5. The minimum expected count is 7.24. | | | | | | |
| d. 2 cells (50.0%) have expected count less than 5. The minimum expected count is .38. | | | | | | |
| e. No statistics are computed because Abdominal drain and Clavien-Dindo classification Grade I are constants. | | | | | | |

**Abdominal drain * Clavien-Dindo classification Grade I * Grade III (severe)**

| **Crosstab** | | | | | | |
| --- | --- | --- | --- | --- | --- | --- |
| Grade III (severe) | | | | Clavien-Dindo classification Grade I | | Total |
|  |  |  |  | 0 | 1 |  |
| 0 | Abdominal drain | 0 | Count | 10 | 1 | 11 |
|  |  |  | % within Abdominal drain | 90.9% | 9.1% | 100.0% |
|  |  |  | % within Clavien-Dindo classification Grade I | 33.3% | 100.0% | 35.5% |
|  |  |  | % of Total | 32.3% | 3.2% | 35.5% |
|  |  | 1 | Count | 20 | 0 | 20 |
|  |  |  | % within Abdominal drain | 100.0% | 0.0% | 100.0% |
|  |  |  | % within Clavien-Dindo classification Grade I | 66.7% | 0.0% | 64.5% |
|  |  |  | % of Total | 64.5% | 0.0% | 64.5% |
|  | Total | | Count | 30 | 1 | 31 |
|  |  |  | % within Abdominal drain | 96.8% | 3.2% | 100.0% |
|  |  |  | % within Clavien-Dindo classification Grade I | 100.0% | 100.0% | 100.0% |
|  |  |  | % of Total | 96.8% | 3.2% | 100.0% |
| Total | Abdominal drain | 0 | Count | 10 | 1 | 11 |
|  |  |  | % within Abdominal drain | 90.9% | 9.1% | 100.0% |
|  |  |  | % within Clavien-Dindo classification Grade I | 33.3% | 100.0% | 35.5% |
|  |  |  | % of Total | 32.3% | 3.2% | 35.5% |
|  |  | 1 | Count | 20 | 0 | 20 |
|  |  |  | % within Abdominal drain | 100.0% | 0.0% | 100.0% |
|  |  |  | % within Clavien-Dindo classification Grade I | 66.7% | 0.0% | 64.5% |
|  |  |  | % of Total | 64.5% | 0.0% | 64.5% |
|  | Total | | Count | 30 | 1 | 31 |
|  |  |  | % within Abdominal drain | 96.8% | 3.2% | 100.0% |
|  |  |  | % within Clavien-Dindo classification Grade I | 100.0% | 100.0% | 100.0% |
|  |  |  | % of Total | 96.8% | 3.2% | 100.0% |

| **Chi-Square Tests** | | | | | | |
| --- | --- | --- | --- | --- | --- | --- |
| Grade III (severe) | | Value | df | Asymptotic Significance (2-sided) | Exact Sig. (2-sided) | Exact Sig. (1-sided) |
| 0 | Pearson Chi-Square | 1.879^a^ | 1 | .170 |  |  |
|  | Continuity Correction^b^ | .095 | 1 | .758 |  |  |
|  | Likelihood Ratio | 2.133 | 1 | .144 |  |  |
|  | Fisher's Exact Test |  |  |  | .355 | .355 |
|  | Linear-by-Linear Association | 1.818 | 1 | .178 |  |  |
|  | N of Valid Cases | 31 |  |  |  |  |
| Total | Pearson Chi-Square | 1.879^a^ | 1 | .170 |  |  |
|  | Continuity Correction^b^ | .095 | 1 | .758 |  |  |
|  | Likelihood Ratio | 2.133 | 1 | .144 |  |  |
|  | Fisher's Exact Test |  |  |  | .355 | .355 |
|  | Linear-by-Linear Association | 1.818 | 1 | .178 |  |  |
|  | N of Valid Cases | 31 |  |  |  |  |
| a. 2 cells (50.0%) have expected count less than 5. The minimum expected count is .35. | | | | | | |
| b. Computed only for a 2x2 table | | | | | | |

**Abdominal drain * Grade II * ASA class**

| **Crosstab** | | | | | | |
| --- | --- | --- | --- | --- | --- | --- |
| ASA class | | | | Grade II | | Total |
|  |  |  |  | 0 | 1 |  |
| 1 | Abdominal drain | 0 | Count | 64 | 1 | 65 |
|  |  |  | % within Abdominal drain | 98.5% | 1.5% | 100.0% |
|  |  |  | % within Grade II | 52.0% | 50.0% | 52.0% |
|  |  |  | % of Total | 51.2% | 0.8% | 52.0% |
|  |  | 1 | Count | 59 | 1 | 60 |
|  |  |  | % within Abdominal drain | 98.3% | 1.7% | 100.0% |
|  |  |  | % within Grade II | 48.0% | 50.0% | 48.0% |
|  |  |  | % of Total | 47.2% | 0.8% | 48.0% |
|  | Total | | Count | 123 | 2 | 125 |
|  |  |  | % within Abdominal drain | 98.4% | 1.6% | 100.0% |
|  |  |  | % within Grade II | 100.0% | 100.0% | 100.0% |
|  |  |  | % of Total | 98.4% | 1.6% | 100.0% |
| 2 | Abdominal drain | 0 | Count | 41 | 3 | 44 |
|  |  |  | % within Abdominal drain | 93.2% | 6.8% | 100.0% |
|  |  |  | % within Grade II | 45.1% | 100.0% | 46.8% |
|  |  |  | % of Total | 43.6% | 3.2% | 46.8% |
|  |  | 1 | Count | 50 | 0 | 50 |
|  |  |  | % within Abdominal drain | 100.0% | 0.0% | 100.0% |
|  |  |  | % within Grade II | 54.9% | 0.0% | 53.2% |
|  |  |  | % of Total | 53.2% | 0.0% | 53.2% |
|  | Total | | Count | 91 | 3 | 94 |
|  |  |  | % within Abdominal drain | 96.8% | 3.2% | 100.0% |
|  |  |  | % within Grade II | 100.0% | 100.0% | 100.0% |
|  |  |  | % of Total | 96.8% | 3.2% | 100.0% |
| 3 | Abdominal drain | 0 | Count | 6 |  | 6 |
|  |  |  | % within Abdominal drain | 100.0% |  | 100.0% |
|  |  |  | % within Grade II | 46.2% |  | 46.2% |
|  |  |  | % of Total | 46.2% |  | 46.2% |
|  |  | 1 | Count | 7 |  | 7 |
|  |  |  | % within Abdominal drain | 100.0% |  | 100.0% |
|  |  |  | % within Grade II | 53.8% |  | 53.8% |
|  |  |  | % of Total | 53.8% |  | 53.8% |
|  | Total | | Count | 13 |  | 13 |
|  |  |  | % within Abdominal drain | 100.0% |  | 100.0% |
|  |  |  | % within Grade II | 100.0% |  | 100.0% |
|  |  |  | % of Total | 100.0% |  | 100.0% |
| Total | Abdominal drain | 0 | Count | 111 | 4 | 115 |
|  |  |  | % within Abdominal drain | 96.5% | 3.5% | 100.0% |
|  |  |  | % within Grade II | 48.9% | 80.0% | 49.6% |
|  |  |  | % of Total | 47.8% | 1.7% | 49.6% |
|  |  | 1 | Count | 116 | 1 | 117 |
|  |  |  | % within Abdominal drain | 99.1% | 0.9% | 100.0% |
|  |  |  | % within Grade II | 51.1% | 20.0% | 50.4% |
|  |  |  | % of Total | 50.0% | 0.4% | 50.4% |
|  | Total | | Count | 227 | 5 | 232 |
|  |  |  | % within Abdominal drain | 97.8% | 2.2% | 100.0% |
|  |  |  | % within Grade II | 100.0% | 100.0% | 100.0% |
|  |  |  | % of Total | 97.8% | 2.2% | 100.0% |

| **Chi-Square Tests** | | | | | | |
| --- | --- | --- | --- | --- | --- | --- |
| ASA class | | Value | df | Asymptotic Significance (2-sided) | Exact Sig. (2-sided) | Exact Sig. (1-sided) |
| 1 | Pearson Chi-Square | .003^c^ | 1 | .954 |  |  |
|  | Continuity Correction^b^ | .000 | 1 | 1.000 |  |  |
|  | Likelihood Ratio | .003 | 1 | .955 |  |  |
|  | Fisher's Exact Test |  |  |  | 1.000 | .732 |
|  | Linear-by-Linear Association | .003 | 1 | .955 |  |  |
|  | N of Valid Cases | 125 |  |  |  |  |
| 2 | Pearson Chi-Square | 3.521^d^ | 1 | .061 |  |  |
|  | Continuity Correction^b^ | 1.660 | 1 | .198 |  |  |
|  | Likelihood Ratio | 4.667 | 1 | .031 |  |  |
|  | Fisher's Exact Test |  |  |  | .099 | .099 |
|  | Linear-by-Linear Association | 3.484 | 1 | .062 |  |  |
|  | N of Valid Cases | 94 |  |  |  |  |
| 3 | Pearson Chi-Square | .^e^ |  |  |  |  |
|  | N of Valid Cases | 13 |  |  |  |  |
| Total | Pearson Chi-Square | 1.893^a^ | 1 | .169 |  |  |
|  | Continuity Correction^b^ | .853 | 1 | .356 |  |  |
|  | Likelihood Ratio | 2.020 | 1 | .155 |  |  |
|  | Fisher's Exact Test |  |  |  | .211 | .179 |
|  | Linear-by-Linear Association | 1.885 | 1 | .170 |  |  |
|  | N of Valid Cases | 232 |  |  |  |  |
| a. 2 cells (50.0%) have expected count less than 5. The minimum expected count is 2.48. | | | | | | |
| b. Computed only for a 2x2 table | | | | | | |
| c. 2 cells (50.0%) have expected count less than 5. The minimum expected count is .96. | | | | | | |
| d. 2 cells (50.0%) have expected count less than 5. The minimum expected count is 1.40. | | | | | | |
| e. No statistics are computed because Grade II is a constant. | | | | | | |

**Abdominal drain * Grade II * Diabetes mellitus**

| **Crosstab** | | | | | | |
| --- | --- | --- | --- | --- | --- | --- |
| Diabetes mellitus | | | | Grade II | | Total |
|  |  |  |  | 0 | 1 |  |
| 0 | Abdominal drain | 0 | Count | 107 | 4 | 111 |
|  |  |  | % within Abdominal drain | 96.4% | 3.6% | 100.0% |
|  |  |  | % within Grade II | 50.0% | 80.0% | 50.7% |
|  |  |  | % of Total | 48.9% | 1.8% | 50.7% |
|  |  | 1 | Count | 107 | 1 | 108 |
|  |  |  | % within Abdominal drain | 99.1% | 0.9% | 100.0% |
|  |  |  | % within Grade II | 50.0% | 20.0% | 49.3% |
|  |  |  | % of Total | 48.9% | 0.5% | 49.3% |
|  | Total | | Count | 214 | 5 | 219 |
|  |  |  | % within Abdominal drain | 97.7% | 2.3% | 100.0% |
|  |  |  | % within Grade II | 100.0% | 100.0% | 100.0% |
|  |  |  | % of Total | 97.7% | 2.3% | 100.0% |
| 1 | Abdominal drain | 0 | Count | 4 |  | 4 |
|  |  |  | % within Abdominal drain | 100.0% |  | 100.0% |
|  |  |  | % within Grade II | 30.8% |  | 30.8% |
|  |  |  | % of Total | 30.8% |  | 30.8% |
|  |  | 1 | Count | 9 |  | 9 |
|  |  |  | % within Abdominal drain | 100.0% |  | 100.0% |
|  |  |  | % within Grade II | 69.2% |  | 69.2% |
|  |  |  | % of Total | 69.2% |  | 69.2% |
|  | Total | | Count | 13 |  | 13 |
|  |  |  | % within Abdominal drain | 100.0% |  | 100.0% |
|  |  |  | % within Grade II | 100.0% |  | 100.0% |
|  |  |  | % of Total | 100.0% |  | 100.0% |
| Total | Abdominal drain | 0 | Count | 111 | 4 | 115 |
|  |  |  | % within Abdominal drain | 96.5% | 3.5% | 100.0% |
|  |  |  | % within Grade II | 48.9% | 80.0% | 49.6% |
|  |  |  | % of Total | 47.8% | 1.7% | 49.6% |
|  |  | 1 | Count | 116 | 1 | 117 |
|  |  |  | % within Abdominal drain | 99.1% | 0.9% | 100.0% |
|  |  |  | % within Grade II | 51.1% | 20.0% | 50.4% |
|  |  |  | % of Total | 50.0% | 0.4% | 50.4% |
|  | Total | | Count | 227 | 5 | 232 |
|  |  |  | % within Abdominal drain | 97.8% | 2.2% | 100.0% |
|  |  |  | % within Grade II | 100.0% | 100.0% | 100.0% |
|  |  |  | % of Total | 97.8% | 2.2% | 100.0% |

| **Chi-Square Tests** | | | | | | |
| --- | --- | --- | --- | --- | --- | --- |
| Diabetes mellitus | | Value | df | Asymptotic Significance (2-sided) | Exact Sig. (2-sided) | Exact Sig. (1-sided) |
| 0 | Pearson Chi-Square | 1.759^c^ | 1 | .185 |  |  |
|  | Continuity Correction^b^ | .764 | 1 | .382 |  |  |
|  | Likelihood Ratio | 1.886 | 1 | .170 |  |  |
|  | Fisher's Exact Test |  |  |  | .369 | .193 |
|  | Linear-by-Linear Association | 1.751 | 1 | .186 |  |  |
|  | N of Valid Cases | 219 |  |  |  |  |
| 1 | Pearson Chi-Square | .^d^ |  |  |  |  |
|  | N of Valid Cases | 13 |  |  |  |  |
| Total | Pearson Chi-Square | 1.893^a^ | 1 | .169 |  |  |
|  | Continuity Correction^b^ | .853 | 1 | .356 |  |  |
|  | Likelihood Ratio | 2.020 | 1 | .155 |  |  |
|  | Fisher's Exact Test |  |  |  | .211 | .179 |
|  | Linear-by-Linear Association | 1.885 | 1 | .170 |  |  |
|  | N of Valid Cases | 232 |  |  |  |  |
| a. 2 cells (50.0%) have expected count less than 5. The minimum expected count is 2.48. | | | | | | |
| b. Computed only for a 2x2 table | | | | | | |
| c. 2 cells (50.0%) have expected count less than 5. The minimum expected count is 2.47. | | | | | | |
| d. No statistics are computed because Grade II is a constant. | | | | | | |

**Abdominal drain * Grade II * Active smoker (Yes/No)**

| **Crosstab** | | | | | | |
| --- | --- | --- | --- | --- | --- | --- |
| Active smoker (Yes/No) | | | | Grade II | | Total |
|  |  |  |  | 0 | 1 |  |
| 0 | Abdominal drain | 0 | Count | 85 | 3 | 88 |
|  |  |  | % within Abdominal drain | 96.6% | 3.4% | 100.0% |
|  |  |  | % within Grade II | 48.6% | 75.0% | 49.2% |
|  |  |  | % of Total | 47.5% | 1.7% | 49.2% |
|  |  | 1 | Count | 90 | 1 | 91 |
|  |  |  | % within Abdominal drain | 98.9% | 1.1% | 100.0% |
|  |  |  | % within Grade II | 51.4% | 25.0% | 50.8% |
|  |  |  | % of Total | 50.3% | 0.6% | 50.8% |
|  | Total | | Count | 175 | 4 | 179 |
|  |  |  | % within Abdominal drain | 97.8% | 2.2% | 100.0% |
|  |  |  | % within Grade II | 100.0% | 100.0% | 100.0% |
|  |  |  | % of Total | 97.8% | 2.2% | 100.0% |
| 1 | Abdominal drain | 0 | Count | 26 | 1 | 27 |
|  |  |  | % within Abdominal drain | 96.3% | 3.7% | 100.0% |
|  |  |  | % within Grade II | 50.0% | 100.0% | 50.9% |
|  |  |  | % of Total | 49.1% | 1.9% | 50.9% |
|  |  | 1 | Count | 26 | 0 | 26 |
|  |  |  | % within Abdominal drain | 100.0% | 0.0% | 100.0% |
|  |  |  | % within Grade II | 50.0% | 0.0% | 49.1% |
|  |  |  | % of Total | 49.1% | 0.0% | 49.1% |
|  | Total | | Count | 52 | 1 | 53 |
|  |  |  | % within Abdominal drain | 98.1% | 1.9% | 100.0% |
|  |  |  | % within Grade II | 100.0% | 100.0% | 100.0% |
|  |  |  | % of Total | 98.1% | 1.9% | 100.0% |
| Total | Abdominal drain | 0 | Count | 111 | 4 | 115 |
|  |  |  | % within Abdominal drain | 96.5% | 3.5% | 100.0% |
|  |  |  | % within Grade II | 48.9% | 80.0% | 49.6% |
|  |  |  | % of Total | 47.8% | 1.7% | 49.6% |
|  |  | 1 | Count | 116 | 1 | 117 |
|  |  |  | % within Abdominal drain | 99.1% | 0.9% | 100.0% |
|  |  |  | % within Grade II | 51.1% | 20.0% | 50.4% |
|  |  |  | % of Total | 50.0% | 0.4% | 50.4% |
|  | Total | | Count | 227 | 5 | 232 |
|  |  |  | % within Abdominal drain | 97.8% | 2.2% | 100.0% |
|  |  |  | % within Grade II | 100.0% | 100.0% | 100.0% |
|  |  |  | % of Total | 97.8% | 2.2% | 100.0% |

| **Chi-Square Tests** | | | | | | |
| --- | --- | --- | --- | --- | --- | --- |
| Active smoker (Yes/No) | | Value | df | Asymptotic Significance (2-sided) | Exact Sig. (2-sided) | Exact Sig. (1-sided) |
| 0 | Pearson Chi-Square | 1.093^c^ | 1 | .296 |  |  |
|  | Continuity Correction^b^ | .291 | 1 | .589 |  |  |
|  | Likelihood Ratio | 1.139 | 1 | .286 |  |  |
|  | Fisher's Exact Test |  |  |  | .362 | .298 |
|  | Linear-by-Linear Association | 1.087 | 1 | .297 |  |  |
|  | N of Valid Cases | 179 |  |  |  |  |
| 1 | Pearson Chi-Square | .981^d^ | 1 | .322 |  |  |
|  | Continuity Correction^b^ | .000 | 1 | 1.000 |  |  |
|  | Likelihood Ratio | 1.367 | 1 | .242 |  |  |
|  | Fisher's Exact Test |  |  |  | 1.000 | .509 |
|  | Linear-by-Linear Association | .963 | 1 | .326 |  |  |
|  | N of Valid Cases | 53 |  |  |  |  |
| Total | Pearson Chi-Square | 1.893^a^ | 1 | .169 |  |  |
|  | Continuity Correction^b^ | .853 | 1 | .356 |  |  |
|  | Likelihood Ratio | 2.020 | 1 | .155 |  |  |
|  | Fisher's Exact Test |  |  |  | .211 | .179 |
|  | Linear-by-Linear Association | 1.885 | 1 | .170 |  |  |
|  | N of Valid Cases | 232 |  |  |  |  |
| a. 2 cells (50.0%) have expected count less than 5. The minimum expected count is 2.48. | | | | | | |
| b. Computed only for a 2x2 table | | | | | | |
| c. 2 cells (50.0%) have expected count less than 5. The minimum expected count is 1.97. | | | | | | |
| d. 2 cells (50.0%) have expected count less than 5. The minimum expected count is .49. | | | | | | |

**Abdominal drain * Grade II * Grade I (mild)**

| **Crosstab** | | | | | | |
| --- | --- | --- | --- | --- | --- | --- |
| Grade I (mild) | | | | Grade II | | Total |
|  |  |  |  | 0 | 1 |  |
|  | Abdominal drain | 0 | Count | 102 | 2 | 104 |
|  |  |  | % within Abdominal drain | 98.1% | 1.9% | 100.0% |
|  |  |  | % within Grade II | 51.3% | 100.0% | 51.7% |
|  |  |  | % of Total | 50.7% | 1.0% | 51.7% |
|  |  | 1 | Count | 97 | 0 | 97 |
|  |  |  | % within Abdominal drain | 100.0% | 0.0% | 100.0% |
|  |  |  | % within Grade II | 48.7% | 0.0% | 48.3% |
|  |  |  | % of Total | 48.3% | 0.0% | 48.3% |
|  | Total | | Count | 199 | 2 | 201 |
|  |  |  | % within Abdominal drain | 99.0% | 1.0% | 100.0% |
|  |  |  | % within Grade II | 100.0% | 100.0% | 100.0% |
|  |  |  | % of Total | 99.0% | 1.0% | 100.0% |
| 0 | Abdominal drain | 1 | Count | 1 | 1 | 2 |
|  |  |  | % within Abdominal drain | 50.0% | 50.0% | 100.0% |
|  |  |  | % within Grade II | 100.0% | 100.0% | 100.0% |
|  |  |  | % of Total | 50.0% | 50.0% | 100.0% |
|  | Total | | Count | 1 | 1 | 2 |
|  |  |  | % within Abdominal drain | 50.0% | 50.0% | 100.0% |
|  |  |  | % within Grade II | 100.0% | 100.0% | 100.0% |
|  |  |  | % of Total | 50.0% | 50.0% | 100.0% |
| 1 | Abdominal drain | 0 | Count | 9 | 2 | 11 |
|  |  |  | % within Abdominal drain | 81.8% | 18.2% | 100.0% |
|  |  |  | % within Grade II | 33.3% | 100.0% | 37.9% |
|  |  |  | % of Total | 31.0% | 6.9% | 37.9% |
|  |  | 1 | Count | 18 | 0 | 18 |
|  |  |  | % within Abdominal drain | 100.0% | 0.0% | 100.0% |
|  |  |  | % within Grade II | 66.7% | 0.0% | 62.1% |
|  |  |  | % of Total | 62.1% | 0.0% | 62.1% |
|  | Total | | Count | 27 | 2 | 29 |
|  |  |  | % within Abdominal drain | 93.1% | 6.9% | 100.0% |
|  |  |  | % within Grade II | 100.0% | 100.0% | 100.0% |
|  |  |  | % of Total | 93.1% | 6.9% | 100.0% |
| Total | Abdominal drain | 0 | Count | 111 | 4 | 115 |
|  |  |  | % within Abdominal drain | 96.5% | 3.5% | 100.0% |
|  |  |  | % within Grade II | 48.9% | 80.0% | 49.6% |
|  |  |  | % of Total | 47.8% | 1.7% | 49.6% |
|  |  | 1 | Count | 116 | 1 | 117 |
|  |  |  | % within Abdominal drain | 99.1% | 0.9% | 100.0% |
|  |  |  | % within Grade II | 51.1% | 20.0% | 50.4% |
|  |  |  | % of Total | 50.0% | 0.4% | 50.4% |
|  | Total | | Count | 227 | 5 | 232 |
|  |  |  | % within Abdominal drain | 97.8% | 2.2% | 100.0% |
|  |  |  | % within Grade II | 100.0% | 100.0% | 100.0% |
|  |  |  | % of Total | 97.8% | 2.2% | 100.0% |

| **Chi-Square Tests** | | | | | | |
| --- | --- | --- | --- | --- | --- | --- |
| Grade I (mild) | | Value | df | Asymptotic Significance (2-sided) | Exact Sig. (2-sided) | Exact Sig. (1-sided) |
|  | Pearson Chi-Square | 1.884^c^ | 1 | .170 |  |  |
|  | Continuity Correction^b^ | .438 | 1 | .508 |  |  |
|  | Likelihood Ratio | 2.654 | 1 | .103 |  |  |
|  | Fisher's Exact Test |  |  |  | .498 | .266 |
|  | Linear-by-Linear Association | 1.875 | 1 | .171 |  |  |
|  | N of Valid Cases | 201 |  |  |  |  |
| 0 | Pearson Chi-Square | .^d^ |  |  |  |  |
|  | N of Valid Cases | 2 |  |  |  |  |
| 1 | Pearson Chi-Square | 3.515^e^ | 1 | .061 |  |  |
|  | Continuity Correction^b^ | 1.254 | 1 | .263 |  |  |
|  | Likelihood Ratio | 4.124 | 1 | .042 |  |  |
|  | Fisher's Exact Test |  |  |  | .135 | .135 |
|  | Linear-by-Linear Association | 3.394 | 1 | .065 |  |  |
|  | N of Valid Cases | 29 |  |  |  |  |
| Total | Pearson Chi-Square | 1.893^a^ | 1 | .169 |  |  |
|  | Continuity Correction^b^ | .853 | 1 | .356 |  |  |
|  | Likelihood Ratio | 2.020 | 1 | .155 |  |  |
|  | Fisher's Exact Test |  |  |  | .211 | .179 |
|  | Linear-by-Linear Association | 1.885 | 1 | .170 |  |  |
|  | N of Valid Cases | 232 |  |  |  |  |
| a. 2 cells (50.0%) have expected count less than 5. The minimum expected count is 2.48. | | | | | | |
| b. Computed only for a 2x2 table | | | | | | |
| c. 2 cells (50.0%) have expected count less than 5. The minimum expected count is .97. | | | | | | |
| d. No statistics are computed because Abdominal drain is a constant. | | | | | | |
| e. 2 cells (50.0%) have expected count less than 5. The minimum expected count is .76. | | | | | | |

**Abdominal drain * Grade II * Grade II (moderate)**

| **Crosstab** | | | | | | |
| --- | --- | --- | --- | --- | --- | --- |
| Grade II (moderate) | | | | Grade II | | Total |
|  |  |  |  | 0 | 1 |  |
|  | Abdominal drain | 0 | Count | 102 | 2 | 104 |
|  |  |  | % within Abdominal drain | 98.1% | 1.9% | 100.0% |
|  |  |  | % within Grade II | 51.3% | 100.0% | 51.7% |
|  |  |  | % of Total | 50.7% | 1.0% | 51.7% |
|  |  | 1 | Count | 97 | 0 | 97 |
|  |  |  | % within Abdominal drain | 100.0% | 0.0% | 100.0% |
|  |  |  | % within Grade II | 48.7% | 0.0% | 48.3% |
|  |  |  | % of Total | 48.3% | 0.0% | 48.3% |
|  | Total | | Count | 199 | 2 | 201 |
|  |  |  | % within Abdominal drain | 99.0% | 1.0% | 100.0% |
|  |  |  | % within Grade II | 100.0% | 100.0% | 100.0% |
|  |  |  | % of Total | 99.0% | 1.0% | 100.0% |
| 0 | Abdominal drain | 0 | Count | 9 | 2 | 11 |
|  |  |  | % within Abdominal drain | 81.8% | 18.2% | 100.0% |
|  |  |  | % within Grade II | 33.3% | 100.0% | 37.9% |
|  |  |  | % of Total | 31.0% | 6.9% | 37.9% |
|  |  | 1 | Count | 18 | 0 | 18 |
|  |  |  | % within Abdominal drain | 100.0% | 0.0% | 100.0% |
|  |  |  | % within Grade II | 66.7% | 0.0% | 62.1% |
|  |  |  | % of Total | 62.1% | 0.0% | 62.1% |
|  | Total | | Count | 27 | 2 | 29 |
|  |  |  | % within Abdominal drain | 93.1% | 6.9% | 100.0% |
|  |  |  | % within Grade II | 100.0% | 100.0% | 100.0% |
|  |  |  | % of Total | 93.1% | 6.9% | 100.0% |
| 1 | Abdominal drain | 1 | Count | 1 | 1 | 2 |
|  |  |  | % within Abdominal drain | 50.0% | 50.0% | 100.0% |
|  |  |  | % within Grade II | 100.0% | 100.0% | 100.0% |
|  |  |  | % of Total | 50.0% | 50.0% | 100.0% |
|  | Total | | Count | 1 | 1 | 2 |
|  |  |  | % within Abdominal drain | 50.0% | 50.0% | 100.0% |
|  |  |  | % within Grade II | 100.0% | 100.0% | 100.0% |
|  |  |  | % of Total | 50.0% | 50.0% | 100.0% |
| Total | Abdominal drain | 0 | Count | 111 | 4 | 115 |
|  |  |  | % within Abdominal drain | 96.5% | 3.5% | 100.0% |
|  |  |  | % within Grade II | 48.9% | 80.0% | 49.6% |
|  |  |  | % of Total | 47.8% | 1.7% | 49.6% |
|  |  | 1 | Count | 116 | 1 | 117 |
|  |  |  | % within Abdominal drain | 99.1% | 0.9% | 100.0% |
|  |  |  | % within Grade II | 51.1% | 20.0% | 50.4% |
|  |  |  | % of Total | 50.0% | 0.4% | 50.4% |
|  | Total | | Count | 227 | 5 | 232 |
|  |  |  | % within Abdominal drain | 97.8% | 2.2% | 100.0% |
|  |  |  | % within Grade II | 100.0% | 100.0% | 100.0% |
|  |  |  | % of Total | 97.8% | 2.2% | 100.0% |

| **Chi-Square Tests** | | | | | | |
| --- | --- | --- | --- | --- | --- | --- |
| Grade II (moderate) | | Value | df | Asymptotic Significance (2-sided) | Exact Sig. (2-sided) | Exact Sig. (1-sided) |
|  | Pearson Chi-Square | 1.884^c^ | 1 | .170 |  |  |
|  | Continuity Correction^b^ | .438 | 1 | .508 |  |  |
|  | Likelihood Ratio | 2.654 | 1 | .103 |  |  |
|  | Fisher's Exact Test |  |  |  | .498 | .266 |
|  | Linear-by-Linear Association | 1.875 | 1 | .171 |  |  |
|  | N of Valid Cases | 201 |  |  |  |  |
| 0 | Pearson Chi-Square | 3.515^d^ | 1 | .061 |  |  |
|  | Continuity Correction^b^ | 1.254 | 1 | .263 |  |  |
|  | Likelihood Ratio | 4.124 | 1 | .042 |  |  |
|  | Fisher's Exact Test |  |  |  | .135 | .135 |
|  | Linear-by-Linear Association | 3.394 | 1 | .065 |  |  |
|  | N of Valid Cases | 29 |  |  |  |  |
| 1 | Pearson Chi-Square | .^e^ |  |  |  |  |
|  | N of Valid Cases | 2 |  |  |  |  |
| Total | Pearson Chi-Square | 1.893^a^ | 1 | .169 |  |  |
|  | Continuity Correction^b^ | .853 | 1 | .356 |  |  |
|  | Likelihood Ratio | 2.020 | 1 | .155 |  |  |
|  | Fisher's Exact Test |  |  |  | .211 | .179 |
|  | Linear-by-Linear Association | 1.885 | 1 | .170 |  |  |
|  | N of Valid Cases | 232 |  |  |  |  |
| a. 2 cells (50.0%) have expected count less than 5. The minimum expected count is 2.48. | | | | | | |
| b. Computed only for a 2x2 table | | | | | | |
| c. 2 cells (50.0%) have expected count less than 5. The minimum expected count is .97. | | | | | | |
| d. 2 cells (50.0%) have expected count less than 5. The minimum expected count is .76. | | | | | | |
| e. No statistics are computed because Abdominal drain is a constant. | | | | | | |

**Abdominal drain * Grade II * Grade III (severe)**

| **Crosstab** | | | | | | |
| --- | --- | --- | --- | --- | --- | --- |
| Grade III (severe) | | | | Grade II | | Total |
|  |  |  |  | 0 | 1 |  |
| 0 | Abdominal drain | 0 | Count | 9 | 2 | 11 |
|  |  |  | % within Abdominal drain | 81.8% | 18.2% | 100.0% |
|  |  |  | % within Grade II | 32.1% | 66.7% | 35.5% |
|  |  |  | % of Total | 29.0% | 6.5% | 35.5% |
|  |  | 1 | Count | 19 | 1 | 20 |
|  |  |  | % within Abdominal drain | 95.0% | 5.0% | 100.0% |
|  |  |  | % within Grade II | 67.9% | 33.3% | 64.5% |
|  |  |  | % of Total | 61.3% | 3.2% | 64.5% |
|  | Total | | Count | 28 | 3 | 31 |
|  |  |  | % within Abdominal drain | 90.3% | 9.7% | 100.0% |
|  |  |  | % within Grade II | 100.0% | 100.0% | 100.0% |
|  |  |  | % of Total | 90.3% | 9.7% | 100.0% |
| Total | Abdominal drain | 0 | Count | 9 | 2 | 11 |
|  |  |  | % within Abdominal drain | 81.8% | 18.2% | 100.0% |
|  |  |  | % within Grade II | 32.1% | 66.7% | 35.5% |
|  |  |  | % of Total | 29.0% | 6.5% | 35.5% |
|  |  | 1 | Count | 19 | 1 | 20 |
|  |  |  | % within Abdominal drain | 95.0% | 5.0% | 100.0% |
|  |  |  | % within Grade II | 67.9% | 33.3% | 64.5% |
|  |  |  | % of Total | 61.3% | 3.2% | 64.5% |
|  | Total | | Count | 28 | 3 | 31 |
|  |  |  | % within Abdominal drain | 90.3% | 9.7% | 100.0% |
|  |  |  | % within Grade II | 100.0% | 100.0% | 100.0% |
|  |  |  | % of Total | 90.3% | 9.7% | 100.0% |

| **Chi-Square Tests** | | | | | | |
| --- | --- | --- | --- | --- | --- | --- |
| Grade III (severe) | | Value | df | Asymptotic Significance (2-sided) | Exact Sig. (2-sided) | Exact Sig. (1-sided) |
| 0 | Pearson Chi-Square | 1.411^a^ | 1 | .235 |  |  |
|  | Continuity Correction^b^ | .306 | 1 | .580 |  |  |
|  | Likelihood Ratio | 1.340 | 1 | .247 |  |  |
|  | Fisher's Exact Test |  |  |  | .281 | .281 |
|  | Linear-by-Linear Association | 1.365 | 1 | .243 |  |  |
|  | N of Valid Cases | 31 |  |  |  |  |
| Total | Pearson Chi-Square | 1.411^a^ | 1 | .235 |  |  |
|  | Continuity Correction^b^ | .306 | 1 | .580 |  |  |
|  | Likelihood Ratio | 1.340 | 1 | .247 |  |  |
|  | Fisher's Exact Test |  |  |  | .281 | .281 |
|  | Linear-by-Linear Association | 1.365 | 1 | .243 |  |  |
|  | N of Valid Cases | 31 |  |  |  |  |
| a. 2 cells (50.0%) have expected count less than 5. The minimum expected count is 1.06. | | | | | | |
| b. Computed only for a 2x2 table | | | | | | |

**Abdominal drain * Grade III * ASA class**

| **Crosstab** | | | | | | |
| --- | --- | --- | --- | --- | --- | --- |
| ASA class | | | | Grade III | | Total |
|  |  |  |  | 0 | 1 |  |
| 1 | Abdominal drain | 0 | Count | 65 | 0 | 65 |
|  |  |  | % within Abdominal drain | 100.0% | 0.0% | 100.0% |
|  |  |  | % within Grade III | 52.4% | 0.0% | 52.0% |
|  |  |  | % of Total | 52.0% | 0.0% | 52.0% |
|  |  | 1 | Count | 59 | 1 | 60 |
|  |  |  | % within Abdominal drain | 98.3% | 1.7% | 100.0% |
|  |  |  | % within Grade III | 47.6% | 100.0% | 48.0% |
|  |  |  | % of Total | 47.2% | 0.8% | 48.0% |
|  | Total | | Count | 124 | 1 | 125 |
|  |  |  | % within Abdominal drain | 99.2% | 0.8% | 100.0% |
|  |  |  | % within Grade III | 100.0% | 100.0% | 100.0% |
|  |  |  | % of Total | 99.2% | 0.8% | 100.0% |
| 2 | Abdominal drain | 0 | Count | 44 | 0 | 44 |
|  |  |  | % within Abdominal drain | 100.0% | 0.0% | 100.0% |
|  |  |  | % within Grade III | 47.3% | 0.0% | 46.8% |
|  |  |  | % of Total | 46.8% | 0.0% | 46.8% |
|  |  | 1 | Count | 49 | 1 | 50 |
|  |  |  | % within Abdominal drain | 98.0% | 2.0% | 100.0% |
|  |  |  | % within Grade III | 52.7% | 100.0% | 53.2% |
|  |  |  | % of Total | 52.1% | 1.1% | 53.2% |
|  | Total | | Count | 93 | 1 | 94 |
|  |  |  | % within Abdominal drain | 98.9% | 1.1% | 100.0% |
|  |  |  | % within Grade III | 100.0% | 100.0% | 100.0% |
|  |  |  | % of Total | 98.9% | 1.1% | 100.0% |
| 3 | Abdominal drain | 0 | Count | 6 |  | 6 |
|  |  |  | % within Abdominal drain | 100.0% |  | 100.0% |
|  |  |  | % within Grade III | 46.2% |  | 46.2% |
|  |  |  | % of Total | 46.2% |  | 46.2% |
|  |  | 1 | Count | 7 |  | 7 |
|  |  |  | % within Abdominal drain | 100.0% |  | 100.0% |
|  |  |  | % within Grade III | 53.8% |  | 53.8% |
|  |  |  | % of Total | 53.8% |  | 53.8% |
|  | Total | | Count | 13 |  | 13 |
|  |  |  | % within Abdominal drain | 100.0% |  | 100.0% |
|  |  |  | % within Grade III | 100.0% |  | 100.0% |
|  |  |  | % of Total | 100.0% |  | 100.0% |
| Total | Abdominal drain | 0 | Count | 115 | 0 | 115 |
|  |  |  | % within Abdominal drain | 100.0% | 0.0% | 100.0% |
|  |  |  | % within Grade III | 50.0% | 0.0% | 49.6% |
|  |  |  | % of Total | 49.6% | 0.0% | 49.6% |
|  |  | 1 | Count | 115 | 2 | 117 |
|  |  |  | % within Abdominal drain | 98.3% | 1.7% | 100.0% |
|  |  |  | % within Grade III | 50.0% | 100.0% | 50.4% |
|  |  |  | % of Total | 49.6% | 0.9% | 50.4% |
|  | Total | | Count | 230 | 2 | 232 |
|  |  |  | % within Abdominal drain | 99.1% | 0.9% | 100.0% |
|  |  |  | % within Grade III | 100.0% | 100.0% | 100.0% |
|  |  |  | % of Total | 99.1% | 0.9% | 100.0% |

| **Chi-Square Tests** | | | | | | |
| --- | --- | --- | --- | --- | --- | --- |
| ASA class | | Value | df | Asymptotic Significance (2-sided) | Exact Sig. (2-sided) | Exact Sig. (1-sided) |
| 1 | Pearson Chi-Square | 1.092^c^ | 1 | .296 |  |  |
|  | Continuity Correction^b^ | .002 | 1 | .968 |  |  |
|  | Likelihood Ratio | 1.477 | 1 | .224 |  |  |
|  | Fisher's Exact Test |  |  |  | .480 | .480 |
|  | Linear-by-Linear Association | 1.083 | 1 | .298 |  |  |
|  | N of Valid Cases | 125 |  |  |  |  |
| 2 | Pearson Chi-Square | .889^d^ | 1 | .346 |  |  |
|  | Continuity Correction^b^ | .000 | 1 | 1.000 |  |  |
|  | Likelihood Ratio | 1.272 | 1 | .259 |  |  |
|  | Fisher's Exact Test |  |  |  | 1.000 | .532 |
|  | Linear-by-Linear Association | .880 | 1 | .348 |  |  |
|  | N of Valid Cases | 94 |  |  |  |  |
| 3 | Pearson Chi-Square | .^e^ |  |  |  |  |
|  | N of Valid Cases | 13 |  |  |  |  |
| Total | Pearson Chi-Square | 1.983^a^ | 1 | .159 |  |  |
|  | Continuity Correction^b^ | .487 | 1 | .485 |  |  |
|  | Likelihood Ratio | 2.755 | 1 | .097 |  |  |
|  | Fisher's Exact Test |  |  |  | .498 | .253 |
|  | Linear-by-Linear Association | 1.974 | 1 | .160 |  |  |
|  | N of Valid Cases | 232 |  |  |  |  |
| a. 2 cells (50.0%) have expected count less than 5. The minimum expected count is .99. | | | | | | |
| b. Computed only for a 2x2 table | | | | | | |
| c. 2 cells (50.0%) have expected count less than 5. The minimum expected count is .48. | | | | | | |
| d. 2 cells (50.0%) have expected count less than 5. The minimum expected count is .47. | | | | | | |
| e. No statistics are computed because Grade III is a constant. | | | | | | |

**Abdominal drain * Grade III * Diabetes mellitus**

| **Crosstab** | | | | | | |
| --- | --- | --- | --- | --- | --- | --- |
| Diabetes mellitus | | | | Grade III | | Total |
|  |  |  |  | 0 | 1 |  |
| 0 | Abdominal drain | 0 | Count | 111 | 0 | 111 |
|  |  |  | % within Abdominal drain | 100.0% | 0.0% | 100.0% |
|  |  |  | % within Grade III | 51.2% | 0.0% | 50.7% |
|  |  |  | % of Total | 50.7% | 0.0% | 50.7% |
|  |  | 1 | Count | 106 | 2 | 108 |
|  |  |  | % within Abdominal drain | 98.1% | 1.9% | 100.0% |
|  |  |  | % within Grade III | 48.8% | 100.0% | 49.3% |
|  |  |  | % of Total | 48.4% | 0.9% | 49.3% |
|  | Total | | Count | 217 | 2 | 219 |
|  |  |  | % within Abdominal drain | 99.1% | 0.9% | 100.0% |
|  |  |  | % within Grade III | 100.0% | 100.0% | 100.0% |
|  |  |  | % of Total | 99.1% | 0.9% | 100.0% |
| 1 | Abdominal drain | 0 | Count | 4 |  | 4 |
|  |  |  | % within Abdominal drain | 100.0% |  | 100.0% |
|  |  |  | % within Grade III | 30.8% |  | 30.8% |
|  |  |  | % of Total | 30.8% |  | 30.8% |
|  |  | 1 | Count | 9 |  | 9 |
|  |  |  | % within Abdominal drain | 100.0% |  | 100.0% |
|  |  |  | % within Grade III | 69.2% |  | 69.2% |
|  |  |  | % of Total | 69.2% |  | 69.2% |
|  | Total | | Count | 13 |  | 13 |
|  |  |  | % within Abdominal drain | 100.0% |  | 100.0% |
|  |  |  | % within Grade III | 100.0% |  | 100.0% |
|  |  |  | % of Total | 100.0% |  | 100.0% |
| Total | Abdominal drain | 0 | Count | 115 | 0 | 115 |
|  |  |  | % within Abdominal drain | 100.0% | 0.0% | 100.0% |
|  |  |  | % within Grade III | 50.0% | 0.0% | 49.6% |
|  |  |  | % of Total | 49.6% | 0.0% | 49.6% |
|  |  | 1 | Count | 115 | 2 | 117 |
|  |  |  | % within Abdominal drain | 98.3% | 1.7% | 100.0% |
|  |  |  | % within Grade III | 50.0% | 100.0% | 50.4% |
|  |  |  | % of Total | 49.6% | 0.9% | 50.4% |
|  | Total | | Count | 230 | 2 | 232 |
|  |  |  | % within Abdominal drain | 99.1% | 0.9% | 100.0% |
|  |  |  | % within Grade III | 100.0% | 100.0% | 100.0% |
|  |  |  | % of Total | 99.1% | 0.9% | 100.0% |

| **Chi-Square Tests** | | | | | | |
| --- | --- | --- | --- | --- | --- | --- |
| Diabetes mellitus | | Value | df | Asymptotic Significance (2-sided) | Exact Sig. (2-sided) | Exact Sig. (1-sided) |
| 0 | Pearson Chi-Square | 2.075^c^ | 1 | .150 |  |  |
|  | Continuity Correction^b^ | .533 | 1 | .465 |  |  |
|  | Likelihood Ratio | 2.847 | 1 | .092 |  |  |
|  | Fisher's Exact Test |  |  |  | .242 | .242 |
|  | Linear-by-Linear Association | 2.065 | 1 | .151 |  |  |
|  | N of Valid Cases | 219 |  |  |  |  |
| 1 | Pearson Chi-Square | .^d^ |  |  |  |  |
|  | N of Valid Cases | 13 |  |  |  |  |
| Total | Pearson Chi-Square | 1.983^a^ | 1 | .159 |  |  |
|  | Continuity Correction^b^ | .487 | 1 | .485 |  |  |
|  | Likelihood Ratio | 2.755 | 1 | .097 |  |  |
|  | Fisher's Exact Test |  |  |  | .498 | .253 |
|  | Linear-by-Linear Association | 1.974 | 1 | .160 |  |  |
|  | N of Valid Cases | 232 |  |  |  |  |
| a. 2 cells (50.0%) have expected count less than 5. The minimum expected count is .99. | | | | | | |
| b. Computed only for a 2x2 table | | | | | | |
| c. 2 cells (50.0%) have expected count less than 5. The minimum expected count is .99. | | | | | | |
| d. No statistics are computed because Grade III is a constant. | | | | | | |

**Abdominal drain * Grade III * Active smoker (Yes/No)**

| **Crosstab** | | | | | | |
| --- | --- | --- | --- | --- | --- | --- |
| Active smoker (Yes/No) | | | | Grade III | | Total |
|  |  |  |  | 0 | 1 |  |
| 0 | Abdominal drain | 0 | Count | 88 | 0 | 88 |
|  |  |  | % within Abdominal drain | 100.0% | 0.0% | 100.0% |
|  |  |  | % within Grade III | 49.4% | 0.0% | 49.2% |
|  |  |  | % of Total | 49.2% | 0.0% | 49.2% |
|  |  | 1 | Count | 90 | 1 | 91 |
|  |  |  | % within Abdominal drain | 98.9% | 1.1% | 100.0% |
|  |  |  | % within Grade III | 50.6% | 100.0% | 50.8% |
|  |  |  | % of Total | 50.3% | 0.6% | 50.8% |
|  | Total | | Count | 178 | 1 | 179 |
|  |  |  | % within Abdominal drain | 99.4% | 0.6% | 100.0% |
|  |  |  | % within Grade III | 100.0% | 100.0% | 100.0% |
|  |  |  | % of Total | 99.4% | 0.6% | 100.0% |
| 1 | Abdominal drain | 0 | Count | 27 | 0 | 27 |
|  |  |  | % within Abdominal drain | 100.0% | 0.0% | 100.0% |
|  |  |  | % within Grade III | 51.9% | 0.0% | 50.9% |
|  |  |  | % of Total | 50.9% | 0.0% | 50.9% |
|  |  | 1 | Count | 25 | 1 | 26 |
|  |  |  | % within Abdominal drain | 96.2% | 3.8% | 100.0% |
|  |  |  | % within Grade III | 48.1% | 100.0% | 49.1% |
|  |  |  | % of Total | 47.2% | 1.9% | 49.1% |
|  | Total | | Count | 52 | 1 | 53 |
|  |  |  | % within Abdominal drain | 98.1% | 1.9% | 100.0% |
|  |  |  | % within Grade III | 100.0% | 100.0% | 100.0% |
|  |  |  | % of Total | 98.1% | 1.9% | 100.0% |
| Total | Abdominal drain | 0 | Count | 115 | 0 | 115 |
|  |  |  | % within Abdominal drain | 100.0% | 0.0% | 100.0% |
|  |  |  | % within Grade III | 50.0% | 0.0% | 49.6% |
|  |  |  | % of Total | 49.6% | 0.0% | 49.6% |
|  |  | 1 | Count | 115 | 2 | 117 |
|  |  |  | % within Abdominal drain | 98.3% | 1.7% | 100.0% |
|  |  |  | % within Grade III | 50.0% | 100.0% | 50.4% |
|  |  |  | % of Total | 49.6% | 0.9% | 50.4% |
|  | Total | | Count | 230 | 2 | 232 |
|  |  |  | % within Abdominal drain | 99.1% | 0.9% | 100.0% |
|  |  |  | % within Grade III | 100.0% | 100.0% | 100.0% |
|  |  |  | % of Total | 99.1% | 0.9% | 100.0% |

| **Chi-Square Tests** | | | | | | |
| --- | --- | --- | --- | --- | --- | --- |
| Active smoker (Yes/No) | | Value | df | Asymptotic Significance (2-sided) | Exact Sig. (2-sided) | Exact Sig. (1-sided) |
| 0 | Pearson Chi-Square | .972^c^ | 1 | .324 |  |  |
|  | Continuity Correction^b^ | .000 | 1 | 1.000 |  |  |
|  | Likelihood Ratio | 1.358 | 1 | .244 |  |  |
|  | Fisher's Exact Test |  |  |  | 1.000 | .508 |
|  | Linear-by-Linear Association | .967 | 1 | .325 |  |  |
|  | N of Valid Cases | 179 |  |  |  |  |
| 1 | Pearson Chi-Square | 1.058^d^ | 1 | .304 |  |  |
|  | Continuity Correction^b^ | .000 | 1 | .985 |  |  |
|  | Likelihood Ratio | 1.444 | 1 | .229 |  |  |
|  | Fisher's Exact Test |  |  |  | .491 | .491 |
|  | Linear-by-Linear Association | 1.038 | 1 | .308 |  |  |
|  | N of Valid Cases | 53 |  |  |  |  |
| Total | Pearson Chi-Square | 1.983^a^ | 1 | .159 |  |  |
|  | Continuity Correction^b^ | .487 | 1 | .485 |  |  |
|  | Likelihood Ratio | 2.755 | 1 | .097 |  |  |
|  | Fisher's Exact Test |  |  |  | .498 | .253 |
|  | Linear-by-Linear Association | 1.974 | 1 | .160 |  |  |
|  | N of Valid Cases | 232 |  |  |  |  |
| a. 2 cells (50.0%) have expected count less than 5. The minimum expected count is .99. | | | | | | |
| b. Computed only for a 2x2 table | | | | | | |
| c. 2 cells (50.0%) have expected count less than 5. The minimum expected count is .49. | | | | | | |
| d. 2 cells (50.0%) have expected count less than 5. The minimum expected count is .49. | | | | | | |

**Abdominal drain * Grade III * Grade I (mild)**

| **Crosstab** | | | | | | |
| --- | --- | --- | --- | --- | --- | --- |
| Grade I (mild) | | | | Grade III | | Total |
|  |  |  |  | 0 | 1 |  |
|  | Abdominal drain | 0 | Count | 104 | 0 | 104 |
|  |  |  | % within Abdominal drain | 100.0% | 0.0% | 100.0% |
|  |  |  | % within Grade III | 52.0% | 0.0% | 51.7% |
|  |  |  | % of Total | 51.7% | 0.0% | 51.7% |
|  |  | 1 | Count | 96 | 1 | 97 |
|  |  |  | % within Abdominal drain | 99.0% | 1.0% | 100.0% |
|  |  |  | % within Grade III | 48.0% | 100.0% | 48.3% |
|  |  |  | % of Total | 47.8% | 0.5% | 48.3% |
|  | Total | | Count | 200 | 1 | 201 |
|  |  |  | % within Abdominal drain | 99.5% | 0.5% | 100.0% |
|  |  |  | % within Grade III | 100.0% | 100.0% | 100.0% |
|  |  |  | % of Total | 99.5% | 0.5% | 100.0% |
| 0 | Abdominal drain | 1 | Count | 1 | 1 | 2 |
|  |  |  | % within Abdominal drain | 50.0% | 50.0% | 100.0% |
|  |  |  | % within Grade III | 100.0% | 100.0% | 100.0% |
|  |  |  | % of Total | 50.0% | 50.0% | 100.0% |
|  | Total | | Count | 1 | 1 | 2 |
|  |  |  | % within Abdominal drain | 50.0% | 50.0% | 100.0% |
|  |  |  | % within Grade III | 100.0% | 100.0% | 100.0% |
|  |  |  | % of Total | 50.0% | 50.0% | 100.0% |
| 1 | Abdominal drain | 0 | Count | 11 |  | 11 |
|  |  |  | % within Abdominal drain | 100.0% |  | 100.0% |
|  |  |  | % within Grade III | 37.9% |  | 37.9% |
|  |  |  | % of Total | 37.9% |  | 37.9% |
|  |  | 1 | Count | 18 |  | 18 |
|  |  |  | % within Abdominal drain | 100.0% |  | 100.0% |
|  |  |  | % within Grade III | 62.1% |  | 62.1% |
|  |  |  | % of Total | 62.1% |  | 62.1% |
|  | Total | | Count | 29 |  | 29 |
|  |  |  | % within Abdominal drain | 100.0% |  | 100.0% |
|  |  |  | % within Grade III | 100.0% |  | 100.0% |
|  |  |  | % of Total | 100.0% |  | 100.0% |
| Total | Abdominal drain | 0 | Count | 115 | 0 | 115 |
|  |  |  | % within Abdominal drain | 100.0% | 0.0% | 100.0% |
|  |  |  | % within Grade III | 50.0% | 0.0% | 49.6% |
|  |  |  | % of Total | 49.6% | 0.0% | 49.6% |
|  |  | 1 | Count | 115 | 2 | 117 |
|  |  |  | % within Abdominal drain | 98.3% | 1.7% | 100.0% |
|  |  |  | % within Grade III | 50.0% | 100.0% | 50.4% |
|  |  |  | % of Total | 49.6% | 0.9% | 50.4% |
|  | Total | | Count | 230 | 2 | 232 |
|  |  |  | % within Abdominal drain | 99.1% | 0.9% | 100.0% |
|  |  |  | % within Grade III | 100.0% | 100.0% | 100.0% |
|  |  |  | % of Total | 99.1% | 0.9% | 100.0% |

| **Chi-Square Tests** | | | | | | |
| --- | --- | --- | --- | --- | --- | --- |
| Grade I (mild) | | Value | df | Asymptotic Significance (2-sided) | Exact Sig. (2-sided) | Exact Sig. (1-sided) |
|  | Pearson Chi-Square | 1.078^c^ | 1 | .299 |  |  |
|  | Continuity Correction^b^ | .001 | 1 | .972 |  |  |
|  | Likelihood Ratio | 1.463 | 1 | .227 |  |  |
|  | Fisher's Exact Test |  |  |  | .483 | .483 |
|  | Linear-by-Linear Association | 1.072 | 1 | .300 |  |  |
|  | N of Valid Cases | 201 |  |  |  |  |
| 0 | Pearson Chi-Square | .^d^ |  |  |  |  |
|  | N of Valid Cases | 2 |  |  |  |  |
| 1 | Pearson Chi-Square | .^e^ |  |  |  |  |
|  | N of Valid Cases | 29 |  |  |  |  |
| Total | Pearson Chi-Square | 1.983^a^ | 1 | .159 |  |  |
|  | Continuity Correction^b^ | .487 | 1 | .485 |  |  |
|  | Likelihood Ratio | 2.755 | 1 | .097 |  |  |
|  | Fisher's Exact Test |  |  |  | .498 | .253 |
|  | Linear-by-Linear Association | 1.974 | 1 | .160 |  |  |
|  | N of Valid Cases | 232 |  |  |  |  |
| a. 2 cells (50.0%) have expected count less than 5. The minimum expected count is .99. | | | | | | |
| b. Computed only for a 2x2 table | | | | | | |
| c. 2 cells (50.0%) have expected count less than 5. The minimum expected count is .48. | | | | | | |
| d. No statistics are computed because Abdominal drain is a constant. | | | | | | |
| e. No statistics are computed because Grade III is a constant. | | | | | | |

**Abdominal drain * Grade III * Grade II (moderate)**

| **Crosstab** | | | | | | |
| --- | --- | --- | --- | --- | --- | --- |
| Grade II (moderate) | | | | Grade III | | Total |
|  |  |  |  | 0 | 1 |  |
|  | Abdominal drain | 0 | Count | 104 | 0 | 104 |
|  |  |  | % within Abdominal drain | 100.0% | 0.0% | 100.0% |
|  |  |  | % within Grade III | 52.0% | 0.0% | 51.7% |
|  |  |  | % of Total | 51.7% | 0.0% | 51.7% |
|  |  | 1 | Count | 96 | 1 | 97 |
|  |  |  | % within Abdominal drain | 99.0% | 1.0% | 100.0% |
|  |  |  | % within Grade III | 48.0% | 100.0% | 48.3% |
|  |  |  | % of Total | 47.8% | 0.5% | 48.3% |
|  | Total | | Count | 200 | 1 | 201 |
|  |  |  | % within Abdominal drain | 99.5% | 0.5% | 100.0% |
|  |  |  | % within Grade III | 100.0% | 100.0% | 100.0% |
|  |  |  | % of Total | 99.5% | 0.5% | 100.0% |
| 0 | Abdominal drain | 0 | Count | 11 |  | 11 |
|  |  |  | % within Abdominal drain | 100.0% |  | 100.0% |
|  |  |  | % within Grade III | 37.9% |  | 37.9% |
|  |  |  | % of Total | 37.9% |  | 37.9% |
|  |  | 1 | Count | 18 |  | 18 |
|  |  |  | % within Abdominal drain | 100.0% |  | 100.0% |
|  |  |  | % within Grade III | 62.1% |  | 62.1% |
|  |  |  | % of Total | 62.1% |  | 62.1% |
|  | Total | | Count | 29 |  | 29 |
|  |  |  | % within Abdominal drain | 100.0% |  | 100.0% |
|  |  |  | % within Grade III | 100.0% |  | 100.0% |
|  |  |  | % of Total | 100.0% |  | 100.0% |
| 1 | Abdominal drain | 1 | Count | 1 | 1 | 2 |
|  |  |  | % within Abdominal drain | 50.0% | 50.0% | 100.0% |
|  |  |  | % within Grade III | 100.0% | 100.0% | 100.0% |
|  |  |  | % of Total | 50.0% | 50.0% | 100.0% |
|  | Total | | Count | 1 | 1 | 2 |
|  |  |  | % within Abdominal drain | 50.0% | 50.0% | 100.0% |
|  |  |  | % within Grade III | 100.0% | 100.0% | 100.0% |
|  |  |  | % of Total | 50.0% | 50.0% | 100.0% |
| Total | Abdominal drain | 0 | Count | 115 | 0 | 115 |
|  |  |  | % within Abdominal drain | 100.0% | 0.0% | 100.0% |
|  |  |  | % within Grade III | 50.0% | 0.0% | 49.6% |
|  |  |  | % of Total | 49.6% | 0.0% | 49.6% |
|  |  | 1 | Count | 115 | 2 | 117 |
|  |  |  | % within Abdominal drain | 98.3% | 1.7% | 100.0% |
|  |  |  | % within Grade III | 50.0% | 100.0% | 50.4% |
|  |  |  | % of Total | 49.6% | 0.9% | 50.4% |
|  | Total | | Count | 230 | 2 | 232 |
|  |  |  | % within Abdominal drain | 99.1% | 0.9% | 100.0% |
|  |  |  | % within Grade III | 100.0% | 100.0% | 100.0% |
|  |  |  | % of Total | 99.1% | 0.9% | 100.0% |

| **Chi-Square Tests** | | | | | | |
| --- | --- | --- | --- | --- | --- | --- |
| Grade II (moderate) | | Value | df | Asymptotic Significance (2-sided) | Exact Sig. (2-sided) | Exact Sig. (1-sided) |
|  | Pearson Chi-Square | 1.078^c^ | 1 | .299 |  |  |
|  | Continuity Correction^b^ | .001 | 1 | .972 |  |  |
|  | Likelihood Ratio | 1.463 | 1 | .227 |  |  |
|  | Fisher's Exact Test |  |  |  | .483 | .483 |
|  | Linear-by-Linear Association | 1.072 | 1 | .300 |  |  |
|  | N of Valid Cases | 201 |  |  |  |  |
| 0 | Pearson Chi-Square | .^d^ |  |  |  |  |
|  | N of Valid Cases | 29 |  |  |  |  |
| 1 | Pearson Chi-Square | .^e^ |  |  |  |  |
|  | N of Valid Cases | 2 |  |  |  |  |
| Total | Pearson Chi-Square | 1.983^a^ | 1 | .159 |  |  |
|  | Continuity Correction^b^ | .487 | 1 | .485 |  |  |
|  | Likelihood Ratio | 2.755 | 1 | .097 |  |  |
|  | Fisher's Exact Test |  |  |  | .498 | .253 |
|  | Linear-by-Linear Association | 1.974 | 1 | .160 |  |  |
|  | N of Valid Cases | 232 |  |  |  |  |
| a. 2 cells (50.0%) have expected count less than 5. The minimum expected count is .99. | | | | | | |
| b. Computed only for a 2x2 table | | | | | | |
| c. 2 cells (50.0%) have expected count less than 5. The minimum expected count is .48. | | | | | | |
| d. No statistics are computed because Grade III is a constant. | | | | | | |
| e. No statistics are computed because Abdominal drain is a constant. | | | | | | |

**Abdominal drain * Grade III * Grade III (severe)**

| **Crosstab** | | | | | | |
| --- | --- | --- | --- | --- | --- | --- |
| Grade III (severe) | | | | Grade III | | Total |
|  |  |  |  | 0 | 1 |  |
| 0 | Abdominal drain | 0 | Count | 11 | 0 | 11 |
|  |  |  | % within Abdominal drain | 100.0% | 0.0% | 100.0% |
|  |  |  | % within Grade III | 36.7% | 0.0% | 35.5% |
|  |  |  | % of Total | 35.5% | 0.0% | 35.5% |
|  |  | 1 | Count | 19 | 1 | 20 |
|  |  |  | % within Abdominal drain | 95.0% | 5.0% | 100.0% |
|  |  |  | % within Grade III | 63.3% | 100.0% | 64.5% |
|  |  |  | % of Total | 61.3% | 3.2% | 64.5% |
|  | Total | | Count | 30 | 1 | 31 |
|  |  |  | % within Abdominal drain | 96.8% | 3.2% | 100.0% |
|  |  |  | % within Grade III | 100.0% | 100.0% | 100.0% |
|  |  |  | % of Total | 96.8% | 3.2% | 100.0% |
| Total | Abdominal drain | 0 | Count | 11 | 0 | 11 |
|  |  |  | % within Abdominal drain | 100.0% | 0.0% | 100.0% |
|  |  |  | % within Grade III | 36.7% | 0.0% | 35.5% |
|  |  |  | % of Total | 35.5% | 0.0% | 35.5% |
|  |  | 1 | Count | 19 | 1 | 20 |
|  |  |  | % within Abdominal drain | 95.0% | 5.0% | 100.0% |
|  |  |  | % within Grade III | 63.3% | 100.0% | 64.5% |
|  |  |  | % of Total | 61.3% | 3.2% | 64.5% |
|  | Total | | Count | 30 | 1 | 31 |
|  |  |  | % within Abdominal drain | 96.8% | 3.2% | 100.0% |
|  |  |  | % within Grade III | 100.0% | 100.0% | 100.0% |
|  |  |  | % of Total | 96.8% | 3.2% | 100.0% |

| **Chi-Square Tests** | | | | | | |
| --- | --- | --- | --- | --- | --- | --- |
| Grade III (severe) | | Value | df | Asymptotic Significance (2-sided) | Exact Sig. (2-sided) | Exact Sig. (1-sided) |
| 0 | Pearson Chi-Square | .568^a^ | 1 | .451 |  |  |
|  | Continuity Correction^b^ | .000 | 1 | 1.000 |  |  |
|  | Likelihood Ratio | .895 | 1 | .344 |  |  |
|  | Fisher's Exact Test |  |  |  | 1.000 | .645 |
|  | Linear-by-Linear Association | .550 | 1 | .458 |  |  |
|  | N of Valid Cases | 31 |  |  |  |  |
| Total | Pearson Chi-Square | .568^a^ | 1 | .451 |  |  |
|  | Continuity Correction^b^ | .000 | 1 | 1.000 |  |  |
|  | Likelihood Ratio | .895 | 1 | .344 |  |  |
|  | Fisher's Exact Test |  |  |  | 1.000 | .645 |
|  | Linear-by-Linear Association | .550 | 1 | .458 |  |  |
|  | N of Valid Cases | 31 |  |  |  |  |
| a. 2 cells (50.0%) have expected count less than 5. The minimum expected count is .35. | | | | | | |
| b. Computed only for a 2x2 table | | | | | | |

**Abdominal drain * Wound infection * ASA class**

| **Crosstab** | | | | | | |
| --- | --- | --- | --- | --- | --- | --- |
| ASA class | | | | Wound infection | | Total |
|  |  |  |  | 0 | 1 |  |
| 1 | Abdominal drain | 0 | Count | 62 | 3 | 65 |
|  |  |  | % within Abdominal drain | 95.4% | 4.6% | 100.0% |
|  |  |  | % within Wound infection | 51.7% | 60.0% | 52.0% |
|  |  |  | % of Total | 49.6% | 2.4% | 52.0% |
|  |  | 1 | Count | 58 | 2 | 60 |
|  |  |  | % within Abdominal drain | 96.7% | 3.3% | 100.0% |
|  |  |  | % within Wound infection | 48.3% | 40.0% | 48.0% |
|  |  |  | % of Total | 46.4% | 1.6% | 48.0% |
|  | Total | | Count | 120 | 5 | 125 |
|  |  |  | % within Abdominal drain | 96.0% | 4.0% | 100.0% |
|  |  |  | % within Wound infection | 100.0% | 100.0% | 100.0% |
|  |  |  | % of Total | 96.0% | 4.0% | 100.0% |
| 2 | Abdominal drain | 0 | Count | 38 | 6 | 44 |
|  |  |  | % within Abdominal drain | 86.4% | 13.6% | 100.0% |
|  |  |  | % within Wound infection | 44.2% | 75.0% | 46.8% |
|  |  |  | % of Total | 40.4% | 6.4% | 46.8% |
|  |  | 1 | Count | 48 | 2 | 50 |
|  |  |  | % within Abdominal drain | 96.0% | 4.0% | 100.0% |
|  |  |  | % within Wound infection | 55.8% | 25.0% | 53.2% |
|  |  |  | % of Total | 51.1% | 2.1% | 53.2% |
|  | Total | | Count | 86 | 8 | 94 |
|  |  |  | % within Abdominal drain | 91.5% | 8.5% | 100.0% |
|  |  |  | % within Wound infection | 100.0% | 100.0% | 100.0% |
|  |  |  | % of Total | 91.5% | 8.5% | 100.0% |
| 3 | Abdominal drain | 0 | Count | 6 |  | 6 |
|  |  |  | % within Abdominal drain | 100.0% |  | 100.0% |
|  |  |  | % within Wound infection | 46.2% |  | 46.2% |
|  |  |  | % of Total | 46.2% |  | 46.2% |
|  |  | 1 | Count | 7 |  | 7 |
|  |  |  | % within Abdominal drain | 100.0% |  | 100.0% |
|  |  |  | % within Wound infection | 53.8% |  | 53.8% |
|  |  |  | % of Total | 53.8% |  | 53.8% |
|  | Total | | Count | 13 |  | 13 |
|  |  |  | % within Abdominal drain | 100.0% |  | 100.0% |
|  |  |  | % within Wound infection | 100.0% |  | 100.0% |
|  |  |  | % of Total | 100.0% |  | 100.0% |
| Total | Abdominal drain | 0 | Count | 106 | 9 | 115 |
|  |  |  | % within Abdominal drain | 92.2% | 7.8% | 100.0% |
|  |  |  | % within Wound infection | 48.4% | 69.2% | 49.6% |
|  |  |  | % of Total | 45.7% | 3.9% | 49.6% |
|  |  | 1 | Count | 113 | 4 | 117 |
|  |  |  | % within Abdominal drain | 96.6% | 3.4% | 100.0% |
|  |  |  | % within Wound infection | 51.6% | 30.8% | 50.4% |
|  |  |  | % of Total | 48.7% | 1.7% | 50.4% |
|  | Total | | Count | 219 | 13 | 232 |
|  |  |  | % within Abdominal drain | 94.4% | 5.6% | 100.0% |
|  |  |  | % within Wound infection | 100.0% | 100.0% | 100.0% |
|  |  |  | % of Total | 94.4% | 5.6% | 100.0% |

| **Chi-Square Tests** | | | | | | |
| --- | --- | --- | --- | --- | --- | --- |
| ASA class | | Value | df | Asymptotic Significance (2-sided) | Exact Sig. (2-sided) | Exact Sig. (1-sided) |
| 1 | Pearson Chi-Square | .134^c^ | 1 | .715 |  |  |
|  | Continuity Correction^b^ | .000 | 1 | 1.000 |  |  |
|  | Likelihood Ratio | .135 | 1 | .714 |  |  |
|  | Fisher's Exact Test |  |  |  | 1.000 | .538 |
|  | Linear-by-Linear Association | .132 | 1 | .716 |  |  |
|  | N of Valid Cases | 125 |  |  |  |  |
| 2 | Pearson Chi-Square | 2.791^d^ | 1 | .095 |  |  |
|  | Continuity Correction^b^ | 1.691 | 1 | .193 |  |  |
|  | Likelihood Ratio | 2.875 | 1 | .090 |  |  |
|  | Fisher's Exact Test |  |  |  | .141 | .096 |
|  | Linear-by-Linear Association | 2.761 | 1 | .097 |  |  |
|  | N of Valid Cases | 94 |  |  |  |  |
| 3 | Pearson Chi-Square | .^e^ |  |  |  |  |
|  | N of Valid Cases | 13 |  |  |  |  |
| Total | Pearson Chi-Square | 2.130^a^ | 1 | .144 |  |  |
|  | Continuity Correction^b^ | 1.378 | 1 | .240 |  |  |
|  | Likelihood Ratio | 2.180 | 1 | .140 |  |  |
|  | Fisher's Exact Test |  |  |  | .164 | .120 |
|  | Linear-by-Linear Association | 2.121 | 1 | .145 |  |  |
|  | N of Valid Cases | 232 |  |  |  |  |
| a. 0 cells (0.0%) have expected count less than 5. The minimum expected count is 6.44. | | | | | | |
| b. Computed only for a 2x2 table | | | | | | |
| c. 2 cells (50.0%) have expected count less than 5. The minimum expected count is 2.40. | | | | | | |
| d. 2 cells (50.0%) have expected count less than 5. The minimum expected count is 3.74. | | | | | | |
| e. No statistics are computed because Wound infection is a constant. | | | | | | |

**Abdominal drain * Wound infection * Diabetes mellitus**

| **Crosstab** | | | | | | |
| --- | --- | --- | --- | --- | --- | --- |
| Diabetes mellitus | | | | Wound infection | | Total |
|  |  |  |  | 0 | 1 |  |
| 0 | Abdominal drain | 0 | Count | 102 | 9 | 111 |
|  |  |  | % within Abdominal drain | 91.9% | 8.1% | 100.0% |
|  |  |  | % within Wound infection | 49.5% | 69.2% | 50.7% |
|  |  |  | % of Total | 46.6% | 4.1% | 50.7% |
|  |  | 1 | Count | 104 | 4 | 108 |
|  |  |  | % within Abdominal drain | 96.3% | 3.7% | 100.0% |
|  |  |  | % within Wound infection | 50.5% | 30.8% | 49.3% |
|  |  |  | % of Total | 47.5% | 1.8% | 49.3% |
|  | Total | | Count | 206 | 13 | 219 |
|  |  |  | % within Abdominal drain | 94.1% | 5.9% | 100.0% |
|  |  |  | % within Wound infection | 100.0% | 100.0% | 100.0% |
|  |  |  | % of Total | 94.1% | 5.9% | 100.0% |
| 1 | Abdominal drain | 0 | Count | 4 |  | 4 |
|  |  |  | % within Abdominal drain | 100.0% |  | 100.0% |
|  |  |  | % within Wound infection | 30.8% |  | 30.8% |
|  |  |  | % of Total | 30.8% |  | 30.8% |
|  |  | 1 | Count | 9 |  | 9 |
|  |  |  | % within Abdominal drain | 100.0% |  | 100.0% |
|  |  |  | % within Wound infection | 69.2% |  | 69.2% |
|  |  |  | % of Total | 69.2% |  | 69.2% |
|  | Total | | Count | 13 |  | 13 |
|  |  |  | % within Abdominal drain | 100.0% |  | 100.0% |
|  |  |  | % within Wound infection | 100.0% |  | 100.0% |
|  |  |  | % of Total | 100.0% |  | 100.0% |
| Total | Abdominal drain | 0 | Count | 106 | 9 | 115 |
|  |  |  | % within Abdominal drain | 92.2% | 7.8% | 100.0% |
|  |  |  | % within Wound infection | 48.4% | 69.2% | 49.6% |
|  |  |  | % of Total | 45.7% | 3.9% | 49.6% |
|  |  | 1 | Count | 113 | 4 | 117 |
|  |  |  | % within Abdominal drain | 96.6% | 3.4% | 100.0% |
|  |  |  | % within Wound infection | 51.6% | 30.8% | 50.4% |
|  |  |  | % of Total | 48.7% | 1.7% | 50.4% |
|  | Total | | Count | 219 | 13 | 232 |
|  |  |  | % within Abdominal drain | 94.4% | 5.6% | 100.0% |
|  |  |  | % within Wound infection | 100.0% | 100.0% | 100.0% |
|  |  |  | % of Total | 94.4% | 5.6% | 100.0% |

| **Chi-Square Tests** | | | | | | |
| --- | --- | --- | --- | --- | --- | --- |
| Diabetes mellitus | | Value | df | Asymptotic Significance (2-sided) | Exact Sig. (2-sided) | Exact Sig. (1-sided) |
| 0 | Pearson Chi-Square | 1.902^c^ | 1 | .168 |  |  |
|  | Continuity Correction^b^ | 1.195 | 1 | .274 |  |  |
|  | Likelihood Ratio | 1.952 | 1 | .162 |  |  |
|  | Fisher's Exact Test |  |  |  | .253 | .137 |
|  | Linear-by-Linear Association | 1.893 | 1 | .169 |  |  |
|  | N of Valid Cases | 219 |  |  |  |  |
| 1 | Pearson Chi-Square | .^d^ |  |  |  |  |
|  | N of Valid Cases | 13 |  |  |  |  |
| Total | Pearson Chi-Square | 2.130^a^ | 1 | .144 |  |  |
|  | Continuity Correction^b^ | 1.378 | 1 | .240 |  |  |
|  | Likelihood Ratio | 2.180 | 1 | .140 |  |  |
|  | Fisher's Exact Test |  |  |  | .164 | .120 |
|  | Linear-by-Linear Association | 2.121 | 1 | .145 |  |  |
|  | N of Valid Cases | 232 |  |  |  |  |
| a. 0 cells (0.0%) have expected count less than 5. The minimum expected count is 6.44. | | | | | | |
| b. Computed only for a 2x2 table | | | | | | |
| c. 0 cells (0.0%) have expected count less than 5. The minimum expected count is 6.41. | | | | | | |
| d. No statistics are computed because Wound infection is a constant. | | | | | | |

**Abdominal drain * Wound infection * Active smoker (Yes/No)**

| **Crosstab** | | | | | | |
| --- | --- | --- | --- | --- | --- | --- |
| Active smoker (Yes/No) | | | | Wound infection | | Total |
|  |  |  |  | 0 | 1 |  |
| 0 | Abdominal drain | 0 | Count | 81 | 7 | 88 |
|  |  |  | % within Abdominal drain | 92.0% | 8.0% | 100.0% |
|  |  |  | % within Wound infection | 47.9% | 70.0% | 49.2% |
|  |  |  | % of Total | 45.3% | 3.9% | 49.2% |
|  |  | 1 | Count | 88 | 3 | 91 |
|  |  |  | % within Abdominal drain | 96.7% | 3.3% | 100.0% |
|  |  |  | % within Wound infection | 52.1% | 30.0% | 50.8% |
|  |  |  | % of Total | 49.2% | 1.7% | 50.8% |
|  | Total | | Count | 169 | 10 | 179 |
|  |  |  | % within Abdominal drain | 94.4% | 5.6% | 100.0% |
|  |  |  | % within Wound infection | 100.0% | 100.0% | 100.0% |
|  |  |  | % of Total | 94.4% | 5.6% | 100.0% |
| 1 | Abdominal drain | 0 | Count | 25 | 2 | 27 |
|  |  |  | % within Abdominal drain | 92.6% | 7.4% | 100.0% |
|  |  |  | % within Wound infection | 50.0% | 66.7% | 50.9% |
|  |  |  | % of Total | 47.2% | 3.8% | 50.9% |
|  |  | 1 | Count | 25 | 1 | 26 |
|  |  |  | % within Abdominal drain | 96.2% | 3.8% | 100.0% |
|  |  |  | % within Wound infection | 50.0% | 33.3% | 49.1% |
|  |  |  | % of Total | 47.2% | 1.9% | 49.1% |
|  | Total | | Count | 50 | 3 | 53 |
|  |  |  | % within Abdominal drain | 94.3% | 5.7% | 100.0% |
|  |  |  | % within Wound infection | 100.0% | 100.0% | 100.0% |
|  |  |  | % of Total | 94.3% | 5.7% | 100.0% |
| Total | Abdominal drain | 0 | Count | 106 | 9 | 115 |
|  |  |  | % within Abdominal drain | 92.2% | 7.8% | 100.0% |
|  |  |  | % within Wound infection | 48.4% | 69.2% | 49.6% |
|  |  |  | % of Total | 45.7% | 3.9% | 49.6% |
|  |  | 1 | Count | 113 | 4 | 117 |
|  |  |  | % within Abdominal drain | 96.6% | 3.4% | 100.0% |
|  |  |  | % within Wound infection | 51.6% | 30.8% | 50.4% |
|  |  |  | % of Total | 48.7% | 1.7% | 50.4% |
|  | Total | | Count | 219 | 13 | 232 |
|  |  |  | % within Abdominal drain | 94.4% | 5.6% | 100.0% |
|  |  |  | % within Wound infection | 100.0% | 100.0% | 100.0% |
|  |  |  | % of Total | 94.4% | 5.6% | 100.0% |

| **Chi-Square Tests** | | | | | | |
| --- | --- | --- | --- | --- | --- | --- |
| Active smoker (Yes/No) | | Value | df | Asymptotic Significance (2-sided) | Exact Sig. (2-sided) | Exact Sig. (1-sided) |
| 0 | Pearson Chi-Square | 1.840^c^ | 1 | .175 |  |  |
|  | Continuity Correction^b^ | 1.063 | 1 | .303 |  |  |
|  | Likelihood Ratio | 1.885 | 1 | .170 |  |  |
|  | Fisher's Exact Test |  |  |  | .207 | .151 |
|  | Linear-by-Linear Association | 1.830 | 1 | .176 |  |  |
|  | N of Valid Cases | 179 |  |  |  |  |
| 1 | Pearson Chi-Square | .315^d^ | 1 | .575 |  |  |
|  | Continuity Correction^b^ | .000 | 1 | 1.000 |  |  |
|  | Likelihood Ratio | .321 | 1 | .571 |  |  |
|  | Fisher's Exact Test |  |  |  | 1.000 | .514 |
|  | Linear-by-Linear Association | .309 | 1 | .579 |  |  |
|  | N of Valid Cases | 53 |  |  |  |  |
| Total | Pearson Chi-Square | 2.130^a^ | 1 | .144 |  |  |
|  | Continuity Correction^b^ | 1.378 | 1 | .240 |  |  |
|  | Likelihood Ratio | 2.180 | 1 | .140 |  |  |
|  | Fisher's Exact Test |  |  |  | .164 | .120 |
|  | Linear-by-Linear Association | 2.121 | 1 | .145 |  |  |
|  | N of Valid Cases | 232 |  |  |  |  |
| a. 0 cells (0.0%) have expected count less than 5. The minimum expected count is 6.44. | | | | | | |
| b. Computed only for a 2x2 table | | | | | | |
| c. 1 cells (25.0%) have expected count less than 5. The minimum expected count is 4.92. | | | | | | |
| d. 2 cells (50.0%) have expected count less than 5. The minimum expected count is 1.47. | | | | | | |

**Abdominal drain * Wound infection * Grade I (mild)**

| **Crosstab** | | | | | | |
| --- | --- | --- | --- | --- | --- | --- |
| Grade I (mild) | | | | Wound infection | | Total |
|  |  |  |  | 0 | 1 |  |
|  | Abdominal drain | 0 | Count | 98 | 6 | 104 |
|  |  |  | % within Abdominal drain | 94.2% | 5.8% | 100.0% |
|  |  |  | % within Wound infection | 51.0% | 66.7% | 51.7% |
|  |  |  | % of Total | 48.8% | 3.0% | 51.7% |
|  |  | 1 | Count | 94 | 3 | 97 |
|  |  |  | % within Abdominal drain | 96.9% | 3.1% | 100.0% |
|  |  |  | % within Wound infection | 49.0% | 33.3% | 48.3% |
|  |  |  | % of Total | 46.8% | 1.5% | 48.3% |
|  | Total | | Count | 192 | 9 | 201 |
|  |  |  | % within Abdominal drain | 95.5% | 4.5% | 100.0% |
|  |  |  | % within Wound infection | 100.0% | 100.0% | 100.0% |
|  |  |  | % of Total | 95.5% | 4.5% | 100.0% |
| 0 | Abdominal drain | 1 | Count | 1 | 1 | 2 |
|  |  |  | % within Abdominal drain | 50.0% | 50.0% | 100.0% |
|  |  |  | % within Wound infection | 100.0% | 100.0% | 100.0% |
|  |  |  | % of Total | 50.0% | 50.0% | 100.0% |
|  | Total | | Count | 1 | 1 | 2 |
|  |  |  | % within Abdominal drain | 50.0% | 50.0% | 100.0% |
|  |  |  | % within Wound infection | 100.0% | 100.0% | 100.0% |
|  |  |  | % of Total | 50.0% | 50.0% | 100.0% |
| 1 | Abdominal drain | 0 | Count | 8 | 3 | 11 |
|  |  |  | % within Abdominal drain | 72.7% | 27.3% | 100.0% |
|  |  |  | % within Wound infection | 30.8% | 100.0% | 37.9% |
|  |  |  | % of Total | 27.6% | 10.3% | 37.9% |
|  |  | 1 | Count | 18 | 0 | 18 |
|  |  |  | % within Abdominal drain | 100.0% | 0.0% | 100.0% |
|  |  |  | % within Wound infection | 69.2% | 0.0% | 62.1% |
|  |  |  | % of Total | 62.1% | 0.0% | 62.1% |
|  | Total | | Count | 26 | 3 | 29 |
|  |  |  | % within Abdominal drain | 89.7% | 10.3% | 100.0% |
|  |  |  | % within Wound infection | 100.0% | 100.0% | 100.0% |
|  |  |  | % of Total | 89.7% | 10.3% | 100.0% |
| Total | Abdominal drain | 0 | Count | 106 | 9 | 115 |
|  |  |  | % within Abdominal drain | 92.2% | 7.8% | 100.0% |
|  |  |  | % within Wound infection | 48.4% | 69.2% | 49.6% |
|  |  |  | % of Total | 45.7% | 3.9% | 49.6% |
|  |  | 1 | Count | 113 | 4 | 117 |
|  |  |  | % within Abdominal drain | 96.6% | 3.4% | 100.0% |
|  |  |  | % within Wound infection | 51.6% | 30.8% | 50.4% |
|  |  |  | % of Total | 48.7% | 1.7% | 50.4% |
|  | Total | | Count | 219 | 13 | 232 |
|  |  |  | % within Abdominal drain | 94.4% | 5.6% | 100.0% |
|  |  |  | % within Wound infection | 100.0% | 100.0% | 100.0% |
|  |  |  | % of Total | 94.4% | 5.6% | 100.0% |

| **Chi-Square Tests** | | | | | | |
| --- | --- | --- | --- | --- | --- | --- |
| Grade I (mild) | | Value | df | Asymptotic Significance (2-sided) | Exact Sig. (2-sided) | Exact Sig. (1-sided) |
|  | Pearson Chi-Square | .841^c^ | 1 | .359 |  |  |
|  | Continuity Correction^b^ | .331 | 1 | .565 |  |  |
|  | Likelihood Ratio | .859 | 1 | .354 |  |  |
|  | Fisher's Exact Test |  |  |  | .500 | .285 |
|  | Linear-by-Linear Association | .836 | 1 | .360 |  |  |
|  | N of Valid Cases | 201 |  |  |  |  |
| 0 | Pearson Chi-Square | .^d^ |  |  |  |  |
|  | N of Valid Cases | 2 |  |  |  |  |
| 1 | Pearson Chi-Square | 5.476^e^ | 1 | .019 |  |  |
|  | Continuity Correction^b^ | 2.930 | 1 | .087 |  |  |
|  | Likelihood Ratio | 6.400 | 1 | .011 |  |  |
|  | Fisher's Exact Test |  |  |  | .045 | .045 |
|  | Linear-by-Linear Association | 5.287 | 1 | .021 |  |  |
|  | N of Valid Cases | 29 |  |  |  |  |
| Total | Pearson Chi-Square | 2.130^a^ | 1 | .144 |  |  |
|  | Continuity Correction^b^ | 1.378 | 1 | .240 |  |  |
|  | Likelihood Ratio | 2.180 | 1 | .140 |  |  |
|  | Fisher's Exact Test |  |  |  | .164 | .120 |
|  | Linear-by-Linear Association | 2.121 | 1 | .145 |  |  |
|  | N of Valid Cases | 232 |  |  |  |  |
| a. 0 cells (0.0%) have expected count less than 5. The minimum expected count is 6.44. | | | | | | |
| b. Computed only for a 2x2 table | | | | | | |
| c. 2 cells (50.0%) have expected count less than 5. The minimum expected count is 4.34. | | | | | | |
| d. No statistics are computed because Abdominal drain is a constant. | | | | | | |
| e. 2 cells (50.0%) have expected count less than 5. The minimum expected count is 1.14. | | | | | | |

**Abdominal drain * Wound infection * Grade II (moderate)**

| **Crosstab** | | | | | | |
| --- | --- | --- | --- | --- | --- | --- |
| Grade II (moderate) | | | | Wound infection | | Total |
|  |  |  |  | 0 | 1 |  |
|  | Abdominal drain | 0 | Count | 98 | 6 | 104 |
|  |  |  | % within Abdominal drain | 94.2% | 5.8% | 100.0% |
|  |  |  | % within Wound infection | 51.0% | 66.7% | 51.7% |
|  |  |  | % of Total | 48.8% | 3.0% | 51.7% |
|  |  | 1 | Count | 94 | 3 | 97 |
|  |  |  | % within Abdominal drain | 96.9% | 3.1% | 100.0% |
|  |  |  | % within Wound infection | 49.0% | 33.3% | 48.3% |
|  |  |  | % of Total | 46.8% | 1.5% | 48.3% |
|  | Total | | Count | 192 | 9 | 201 |
|  |  |  | % within Abdominal drain | 95.5% | 4.5% | 100.0% |
|  |  |  | % within Wound infection | 100.0% | 100.0% | 100.0% |
|  |  |  | % of Total | 95.5% | 4.5% | 100.0% |
| 0 | Abdominal drain | 0 | Count | 8 | 3 | 11 |
|  |  |  | % within Abdominal drain | 72.7% | 27.3% | 100.0% |
|  |  |  | % within Wound infection | 30.8% | 100.0% | 37.9% |
|  |  |  | % of Total | 27.6% | 10.3% | 37.9% |
|  |  | 1 | Count | 18 | 0 | 18 |
|  |  |  | % within Abdominal drain | 100.0% | 0.0% | 100.0% |
|  |  |  | % within Wound infection | 69.2% | 0.0% | 62.1% |
|  |  |  | % of Total | 62.1% | 0.0% | 62.1% |
|  | Total | | Count | 26 | 3 | 29 |
|  |  |  | % within Abdominal drain | 89.7% | 10.3% | 100.0% |
|  |  |  | % within Wound infection | 100.0% | 100.0% | 100.0% |
|  |  |  | % of Total | 89.7% | 10.3% | 100.0% |
| 1 | Abdominal drain | 1 | Count | 1 | 1 | 2 |
|  |  |  | % within Abdominal drain | 50.0% | 50.0% | 100.0% |
|  |  |  | % within Wound infection | 100.0% | 100.0% | 100.0% |
|  |  |  | % of Total | 50.0% | 50.0% | 100.0% |
|  | Total | | Count | 1 | 1 | 2 |
|  |  |  | % within Abdominal drain | 50.0% | 50.0% | 100.0% |
|  |  |  | % within Wound infection | 100.0% | 100.0% | 100.0% |
|  |  |  | % of Total | 50.0% | 50.0% | 100.0% |
| Total | Abdominal drain | 0 | Count | 106 | 9 | 115 |
|  |  |  | % within Abdominal drain | 92.2% | 7.8% | 100.0% |
|  |  |  | % within Wound infection | 48.4% | 69.2% | 49.6% |
|  |  |  | % of Total | 45.7% | 3.9% | 49.6% |
|  |  | 1 | Count | 113 | 4 | 117 |
|  |  |  | % within Abdominal drain | 96.6% | 3.4% | 100.0% |
|  |  |  | % within Wound infection | 51.6% | 30.8% | 50.4% |
|  |  |  | % of Total | 48.7% | 1.7% | 50.4% |
|  | Total | | Count | 219 | 13 | 232 |
|  |  |  | % within Abdominal drain | 94.4% | 5.6% | 100.0% |
|  |  |  | % within Wound infection | 100.0% | 100.0% | 100.0% |
|  |  |  | % of Total | 94.4% | 5.6% | 100.0% |

| **Chi-Square Tests** | | | | | | |
| --- | --- | --- | --- | --- | --- | --- |
| Grade II (moderate) | | Value | df | Asymptotic Significance (2-sided) | Exact Sig. (2-sided) | Exact Sig. (1-sided) |
|  | Pearson Chi-Square | .841^c^ | 1 | .359 |  |  |
|  | Continuity Correction^b^ | .331 | 1 | .565 |  |  |
|  | Likelihood Ratio | .859 | 1 | .354 |  |  |
|  | Fisher's Exact Test |  |  |  | .500 | .285 |
|  | Linear-by-Linear Association | .836 | 1 | .360 |  |  |
|  | N of Valid Cases | 201 |  |  |  |  |
| 0 | Pearson Chi-Square | 5.476^d^ | 1 | .019 |  |  |
|  | Continuity Correction^b^ | 2.930 | 1 | .087 |  |  |
|  | Likelihood Ratio | 6.400 | 1 | .011 |  |  |
|  | Fisher's Exact Test |  |  |  | .045 | .045 |
|  | Linear-by-Linear Association | 5.287 | 1 | .021 |  |  |
|  | N of Valid Cases | 29 |  |  |  |  |
| 1 | Pearson Chi-Square | .^e^ |  |  |  |  |
|  | N of Valid Cases | 2 |  |  |  |  |
| Total | Pearson Chi-Square | 2.130^a^ | 1 | .144 |  |  |
|  | Continuity Correction^b^ | 1.378 | 1 | .240 |  |  |
|  | Likelihood Ratio | 2.180 | 1 | .140 |  |  |
|  | Fisher's Exact Test |  |  |  | .164 | .120 |
|  | Linear-by-Linear Association | 2.121 | 1 | .145 |  |  |
|  | N of Valid Cases | 232 |  |  |  |  |
| a. 0 cells (0.0%) have expected count less than 5. The minimum expected count is 6.44. | | | | | | |
| b. Computed only for a 2x2 table | | | | | | |
| c. 2 cells (50.0%) have expected count less than 5. The minimum expected count is 4.34. | | | | | | |
| d. 2 cells (50.0%) have expected count less than 5. The minimum expected count is 1.14. | | | | | | |
| e. No statistics are computed because Abdominal drain is a constant. | | | | | | |

**Abdominal drain * Wound infection * Grade III (severe)**

| **Crosstab** | | | | | | |
| --- | --- | --- | --- | --- | --- | --- |
| Grade III (severe) | | | | Wound infection | | Total |
|  |  |  |  | 0 | 1 |  |
| 0 | Abdominal drain | 0 | Count | 8 | 3 | 11 |
|  |  |  | % within Abdominal drain | 72.7% | 27.3% | 100.0% |
|  |  |  | % within Wound infection | 29.6% | 75.0% | 35.5% |
|  |  |  | % of Total | 25.8% | 9.7% | 35.5% |
|  |  | 1 | Count | 19 | 1 | 20 |
|  |  |  | % within Abdominal drain | 95.0% | 5.0% | 100.0% |
|  |  |  | % within Wound infection | 70.4% | 25.0% | 64.5% |
|  |  |  | % of Total | 61.3% | 3.2% | 64.5% |
|  | Total | | Count | 27 | 4 | 31 |
|  |  |  | % within Abdominal drain | 87.1% | 12.9% | 100.0% |
|  |  |  | % within Wound infection | 100.0% | 100.0% | 100.0% |
|  |  |  | % of Total | 87.1% | 12.9% | 100.0% |
| Total | Abdominal drain | 0 | Count | 8 | 3 | 11 |
|  |  |  | % within Abdominal drain | 72.7% | 27.3% | 100.0% |
|  |  |  | % within Wound infection | 29.6% | 75.0% | 35.5% |
|  |  |  | % of Total | 25.8% | 9.7% | 35.5% |
|  |  | 1 | Count | 19 | 1 | 20 |
|  |  |  | % within Abdominal drain | 95.0% | 5.0% | 100.0% |
|  |  |  | % within Wound infection | 70.4% | 25.0% | 64.5% |
|  |  |  | % of Total | 61.3% | 3.2% | 64.5% |
|  | Total | | Count | 27 | 4 | 31 |
|  |  |  | % within Abdominal drain | 87.1% | 12.9% | 100.0% |
|  |  |  | % within Wound infection | 100.0% | 100.0% | 100.0% |
|  |  |  | % of Total | 87.1% | 12.9% | 100.0% |

| **Chi-Square Tests** | | | | | | |
| --- | --- | --- | --- | --- | --- | --- |
| Grade III (severe) | | Value | df | Asymptotic Significance (2-sided) | Exact Sig. (2-sided) | Exact Sig. (1-sided) |
| 0 | Pearson Chi-Square | 3.133^a^ | 1 | .077 |  |  |
|  | Continuity Correction^b^ | 1.464 | 1 | .226 |  |  |
|  | Likelihood Ratio | 3.010 | 1 | .083 |  |  |
|  | Fisher's Exact Test |  |  |  | .115 | .115 |
|  | Linear-by-Linear Association | 3.032 | 1 | .082 |  |  |
|  | N of Valid Cases | 31 |  |  |  |  |
| Total | Pearson Chi-Square | 3.133^a^ | 1 | .077 |  |  |
|  | Continuity Correction^b^ | 1.464 | 1 | .226 |  |  |
|  | Likelihood Ratio | 3.010 | 1 | .083 |  |  |
|  | Fisher's Exact Test |  |  |  | .115 | .115 |
|  | Linear-by-Linear Association | 3.032 | 1 | .082 |  |  |
|  | N of Valid Cases | 31 |  |  |  |  |
| a. 2 cells (50.0%) have expected count less than 5. The minimum expected count is 1.42. | | | | | | |
| b. Computed only for a 2x2 table | | | | | | |
